# Exploring Factor Structures Using Variational Autoencoder in Personality Research

**DOI:** 10.3389/fpsyg.2022.863926

**Published:** 2022-08-05

**Authors:** Yufei Huang, Jianqiu Zhang

**Affiliations:** ^1^Department of Medicine, University of Pittsburgh School of Medicine, Pittsburgh, PA, United States; ^2^University of Pittsburgh Medical Center Hillman Cancer Center, Pittsburgh, PA, United States; ^3^Department of Electrical and Computer Engineering, The University of Texas, San Antonio, TX, United States

**Keywords:** non-linear factor analysis, variational auto encoder (VAE), personality trait, artificial intelligence, Big 5 personality factors, HEXACO model of personality, deep learning

## Abstract

An accurate personality model is crucial to many research fields. Most personality models have been constructed using linear factor analysis (LFA). In this paper, we investigate if an effective deep learning tool for factor extraction, the Variational Autoencoder (VAE), can be applied to explore the factor structure of a set of personality variables. To compare VAE with LFA, we applied VAE to an International Personality Item Pool (IPIP) Big 5 dataset and an IPIP HEXACO (Humility-Honesty, Emotionality, Extroversion, Agreeableness, Conscientiousness, Openness) dataset. We found that LFA tends to break factors into ever smaller, yet still significant fractions, when the number of assumed latent factors increases, leading to the need to organize personality variables at the factor level and then the facet level. On the other hand, the factor structure returned by VAE is very stable and VAE only adds noise-like factors after significant factors are found as the number of assumed latent factors increases. VAE reported more stable factors by elevating some facets in the HEXACO scale to the factor level. Since this is a data-driven process that exhausts all stable and significant factors that can be found, it is not necessary to further conduct facet level analysis and it is anticipated that VAE will have broad applications in exploratory factor analysis in personality research.

## Introduction

Linear Factor Analysis (LFA) has enabled the discovery of the most popular personality models, including notably the Big 5 model (Fiske, 1949; Norman, 1963; Costa and McCrae, 1992; Goldberg, 1992) and the HEXACO model (Lee and Ashton, 2004, 2005), which have been extensively utilized to study a wide array of topics, such as personality disorder (Saulsman and Page, 2004; Widiger and Lowe, 2007), academic success (Ziegler et al., 2010; Carthy et al., 2014), leadership (Judge and Bono, 2000; Hassan et al., 2016), relationship satisfaction (O'Meara and South, 2019), job performance (Barrick and Mount, 1991), education outcomes (Noftle and Robins, 2007), and health outcomes (Jerram and Coleman, 1999).

The root of applying LFA for the construction of personality models can be traced back to Galton's lexical hypothesis of personality (Galton, 1884), which assumed that significant individual character differences could be discovered in language. Allport and Odbert applied the lexical approach to investigate personality-related dictionary words. They found approximately 4,500 terms that were considered descriptive of personality traits.

Before high-performance computing was available, Cattell first applied the grouped centroid method in factor analysis (Cattell, 1943) to the list of traits generated by Allport and Odbert. He selected 171 from the list and developed a set of 35 to 40 clusters of words. He eventually settled on 16 personality factors (Cattell et al., 1970) and made his data available to other researchers. After the arrival of high-performance computers, later researchers consistently found a five-factor model (Tupes and Christal, 1961; Norman, 1963). Through the years, the terms used for the five-factor model had changed, and finally, Goldberg coined the term “Big 5” personality model consisting of openness, conscientiousness, extraversion, agreeableness, and neuroticism (Goldberg, 1981).

To eliminate the doubt that LFA methods may heavily influence the discovery process of the Big 5 model, Goldberg tested five methods of factor extraction (principal components, principal factors, alpha-factoring, image-factoring, and maximum-likelihood procedures), each rotated by an orthogonal (varimax) and an oblique (oblimin) algorithm (Goldberg, 1990). He found that procedural variations do not change the five-factor structure, and the factor scores across different methods are highly congruent. In addition, Goldberg and Saucier investigated the relationship between person-descriptive adjective clusters and the Big 5 traits. They concluded that mostly all personality-relevant clusters are not “beyond the Big 5” (Goldberg and Saucier, 1998). Furthermore, it was shown that the Big 5 model is replicable across cultures (McCrae et al., 1998). These findings have cemented the unrivaled popularity of the Big 5 model (Feher and Vernon, 2021).

The other side of Big 5's sustained popularity is that few advances have been made in personality model development (Feher and Vernon, 2021). One exception is the HEXACO model, which applied LFA and the lexical approach to several languages worldwide (Lee and Ashton, 2004, 2005; Ashton and Lee, 2007). A sixth personality factor, Honesty-Humility, consistently showed up in cross-cultural studies, which address the fairness and modesty aspects of personality (Ashton and Lee, 2008a,b). The underlying meaning of some of the factors (agreeableness and emotionality) differs slightly from the Big 5 model (Ashton et al., 2014). The HEXACO model is highly correlated to most existing narrow trait models, such as the Dark Triad models outside of the Big 5 model (Lee and Ashton, 2005; Ashton and Lee, 2007; De Vries et al., 2009).

A critical consideration in factor analysis is how many factors should be extracted. For example, in the IPIP HEXACO dataset that we tested, there could be 8 factors on the scree plot. When we required the eigenvalue to be greater than one, there were 37 factors. The large discrepancy in these criteria makes it impossible to know how many factors should be extracted without examining stability across multiple datasets. Although simulated data can be used to determine the threshold on eigenvalues as in parallel analysis (Horn, 1965), since we do not know the actual distribution of the latent factors and the functions that transforms these factors into the measured personality variables, we cannot simulate a ground truth dataset with a known number of factors in VAE. We observed that as the number of assumed latent factors increases in LFA, bigger factors that contain many personality variables tend to break down into smaller yet significant factors and the fractioning process will not stop until a very large number of latent factors. Yet, these smaller factors are not stable. As a result, established personality models only report a small number of stable factors and only six in the case of HEXACO. However, it has been found that facet-level information must be incorporated in applications (Reynolds and Clark, 2001; Samuel and Widiger, 2008). This indicates that factor-level information is insufficient, yet facet-level research is not entirely data-driven (Goldberg, 1999). As a result, personality scales get frequently revised which is costly for data collection and research.

We can view LFA as a type of unsupervised machine learning (ML) method (Chauhan and Singh, 2018) and we can treat the Big 5 or HEXACO traits as latent generative factors that can be transformed to construct the observable personality variables. We can search in the broader context of unsupervised ML to look for a suitable tool for personality model construction.

In ML, the most recent advances have been driven by Deep Learning (DL) (LeCun et al., 2015; Sengupta et al., 2020). DL methods employ artificial neural networks capable of approximating every function under mild assumptions (Cybenko, 1989; Hornik, 1991). DL had enabled phenomenal technological advancements in computer vision (Krizhevsky et al., 2017), natural language processing (Devlin et al., 2018), autonomous vehicles (Sun et al., 2020), personalization, and recommender systems (Jacobson et al., 2016; Batmaz et al., 2019; Bobadilla et al., 2020), and live translation of languages (Castelvecchi, 2016).

Given such a promise, we have also seen significant growth in applying DL methods for personality traits detection based on data gathered from social media platforms (Liu and Zhu, 2016; Yu and Markov, 2017; Kumar and Gavrilova, 2019; Ahmad et al., 2020; Salminen et al., 2020), vision and language samples (Eddine Bekhouche et al., 2017; Chhabra et al., 2019; Rodriguez et al., 2019; Kim et al., 2020; Ren et al., 2021), handwriting samples (Elngar et al., 2020; Remaida et al., 2020), and mobile-sensing data (Baumeister and Montag, 2019; Spathis et al., 2019). In most of these studies, factors from the Big 5 model are used as labels in the training datasets such that neural networks can be trained to predict the Big 5 traits (Azucar et al., 2018; Bhavya et al., 2020; Mehta et al., 2020; Ren et al., 2021).

Despite these advances, the extent to which DL methods are used for personality model construction has not been extensively conducted. It has motivated us to look for a DL-based non-linear factor analysis tool. In this regard, variational autoencoder (VAE) (Kingma and Welling, 2013; Lopez-Alvis et al., 2020) is a state-of-the-art DL method for unsupervised representation learning.

The first versions of autoencoders emerged over two decades ago (Bourlard and Kamp, 1988; Zemel and Hinton, 1993) and they were primarily used for dimensional reduction initially. They consist of an encoding artificial neural network, which outputs a latent representation of the input data, and a decoding neural network that tries to accurately reconstruct the input data from its latent representation. Very shallow versions of autoencoders (with a small number of middle layer nodes) can reproduce the results of principal component analysis (Baldi and Hornik, 1989).

The VAE is motivated by the more general problem of “obtaining a joint distribution over all input variables through learning a generative model, which simulates how the data is generated in the real world” (Kingma and Welling, 2019). It was designed to find a set of “disentangled, semantically meaningful, statistically independent and causal factors of variation in data,” as the original inventor of VAE described it. VAE differs from traditional autoencoders by imposing restrictions on the distribution of latent variables, which allows it to find independent latent variables (Kingma and Welling, 2013). By taking the sampling step that treats the joint posterior distribution of the latent variables as independent, the algorithm is forced to converge to solutions, in which the latent variables are almost independent. Previous empirical evidence (Burgess et al., 2018) shows that in image processing, these factors can often be tied to an “interpretable” factor. VAE and its variants (Ainsworth et al., 2018; Zhou and Wei, 2020) are more “interpretable” compared to common deep neural networks in this sense. Among various variants of VAE, we have employed the original VAE, which can be considered a special case of beta-VAE (Higgins et al., 2016) because VAE performed the best on the tested datasets.

The VAE does not assume that the observed variables are linear combinations of latent factors plus unique factors as in LFA. Compared to PCA, it also drops the assumption that the generating function of the observed variables is linear. VAE only assumes that the latent variables are Gaussian and independent. In this sense, VAE is closer to PCA than LFA.

Given that the deep neural networks in VAE can be configured to simulate non-linear functions (Cybenko, 1989; Hornik, 1991), it has found applications in many areas that require non-linear modeling of the generative process. For example, it has been applied to non-linear channel equalization (Avi and Burshtein, 2020), 3D mesh models transformation in computer animation (Tan et al., 2018), and fault detection in complex non-linear process controls (Wang et al., 2019). We anticipate that VAE can be applied to find latent and independent personality factors while assuming a non-linear underlying psychological process.

Urban and Bauer (2021) first introduced a deep learning-based variational inference (VI) algorithm that applies an importance-weighted autoencoder (IWAE) for exploratory item factor analysis (IFA) that is computationally efficient even in large datasets with many latent factors. IWAE can recover the 5-factor structure of the Big five model based on a large Big5 dataset. IWAE is very similar to our proposed VAE algorithm except that it sets the output layer to predict the log-likelihood probability of all possible responses on a Likert scale. In contrast, in VAE, we set the output layer to produce a continuous variable. Although it has been established that IWAE-like algorithms can be used for exploring the factor structures of a set of personality variables, however, there are still many unanswered questions. We need to develop new performance measures and factor extracting guidelines to compare the difference between VAE and LFA because VAE does not assume linear data models anymore. Specifically, we need to: (i) Select a stable set of factors across multiple VAE runs; (ii) Compare the accuracy of VAE generated personality models to LFA generated models; (iii) Develop a method for inspecting factor-personality variable association because we cannot rely on factor loadings as in LFA; and (iv) Study the stability of the VAE-generated model across different datasets and regions.

We hypothesize that VAE can do the following: (1) generate personality models that have higher correlations between the input and reconstructed personality variables than LFA, and (2) discover more stable factors than LFA.

We are aware of the limitations of self-reported data in generating useful personality models. However, this research is meant to establish the validity of using VAE as a replacement for LFA for exploratory factor analysis. Due to the scope and complexity involved in combining self-reports and observer reports, we plan to combine both types of data and construct useful personality models using VAE in future research.

### Datasets

In this study, we want to compare the performance of VAE-generated models to that of LFA-generated models. We selected two datasets collected based on the two most popular LFA constructed models, the Big 5 and the HEXACO models. Note that this is an initial study on the applicability of VAE to personality model analysis.

### The International Personality Inventory Pool (IPIP) Big 5 Dataset

The IPIP Big 5 factor markers consist of a 50 or a 100-item inventory(Goldberg and Others 2001). We used the 50-item version consisting of 10 items for each of the Big 5 personality factors: Extraversion (E), Agreeableness (A), Conscientiousness (C), Neuroticism (N), and Openness/Intellect (I). Each item is given in a sentence form (e.g., “I am the life of the party”). Participants were requested to read each of the 50 items and then rate on a 5-point scale (from strongly disagree to strongly agree). The dataset was collected through an online questionnaire downloadable at the Open-Source Psychometrics Project (Goettfert and Kriner, n.d.). The dataset contains 19,719 samples, and we have used all samples in our study. The alpha reliability of the factors ranged from 0.80 to 0.89, and the mean and the standard deviation (SD) of the factors are consistent with previous publications (Costa and McCrae, 1992; Goldberg, 1992).

### The IPIP HEXACO Dataset

We downloaded an IPIP HEXACO dataset collected from a questionnaire that measures 240 personality variables from the Open-Source Psychometrics Project website (Goettfert and Kriner, n.d.). The IPIP HEXACO inventory was constructed by correlating all 2036 IPIP items with the 24 HEXACO-Personality Inventory (PI) facet scales (Lee and Ashton, 2004): Honesty-Humility (H) with facets: Sincerity (HSinc), Fairness (HFair), Greed (HGree), Avoidance (HAvoi), Modesty (HMode); Emotionality (E) with facets: Fearfulness (EFear), Anxiety (EAnxi), Dependence (EDepe), Sentimentality (ESent); Extraversion (X) with facets: Social Self-Esteem (XExper), Social Boldness (XSocB), Sociability (XSoci), Liveliness (XLive); Agreeableness (A) with facets Forgivingness (AForg), Gentleness (Agent), Flexibility (AFlex), Patience (APati); Conscientiousness (C) with facets: Organization (COrga), Diligence (CDili), Perfectionism (CPerf), Prudence (CPrud); Openness to Experience (O) with facets: Aesthetic Appreciation (OAesA), Inquisitiveness (OInqu), Creativity (OCrea), and Unconventionality (OUnco).

Within each of these 24 groups of IPIP items, the 10 personality variables showing the highest absolute correlations with their corresponding HEXACO-PI scale were selected. The resulting set of 24 IPIP—HEXACO scales showed alpha reliabilities ranging from 0.73 to 0.88 with a mean of 0.81. Some personality variables were subsequently adjusted to reduce the correlation between Agreeableness and Honesty-Humility items (Ashton et al., 2007).

The IPIP HEXACO dataset contained 22,786 samples. The 240 personality variables were rated on a seven-point scale (1 = strongly disagree, 2 = disagree, 3 = slightly disagree, 4 = neutral, 5 = slightly agree, 6 = agree, and 7 = strongly agree). We kept samples that answered 7 on both verification questions 1 and 2, which were administered at the beginning and the end of the test to ensure that questionnaire takers understood the test and answered all questions as accurately as possible. While lowering the threshold on the validation questions would admit more samples, it resulted in few performance changes. After this filtering process, a total of 18,779 samples were used in our analysis.

## Methods

### Analytical Procedure

Our analytical procedure follows standard protocols in machine learning research. All code is made available on OSF (https://osf.io/6b3w/).

### Data Preprocessing

For all datasets used in the studies, scores from each of the questionnaires are scaled by subtracting the mean and dividing by the SD to shift the distribution to have a mean of zero and a standard deviation of one. This pre-processing step is performed separately for the training and the testing dataset before further processing. Missing values are set to zero after scaling.

### Training VAE Inference and Generative Models

**Figure 1:**
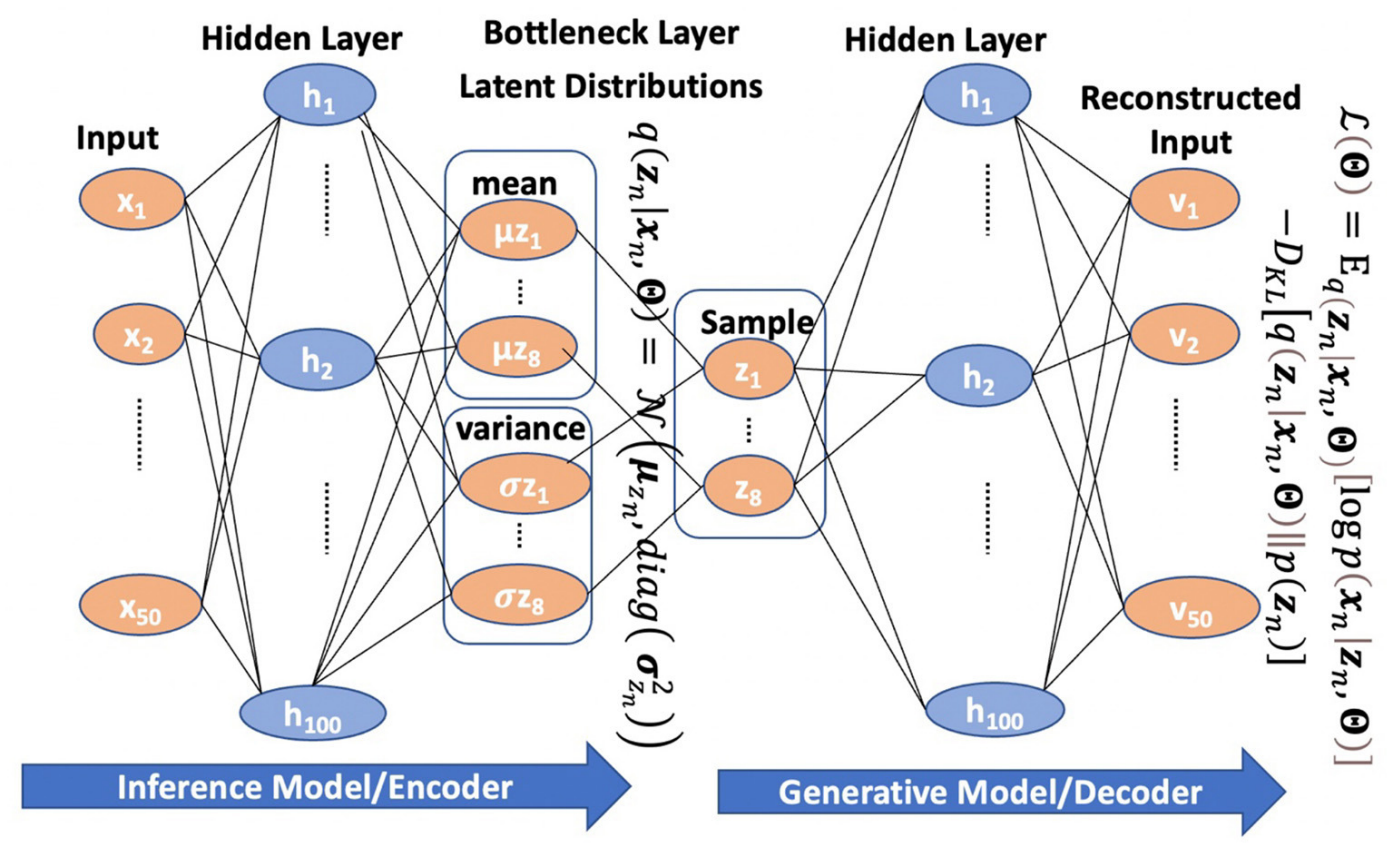
An example of a VAE with 100 hidden middle layer nodes and 8 bottleneck layer nodes.

The VAE is designed to learn interpretable non-linear generative factors. A VAE model comprises two independently parameterized components: an inference model (the encoder) that maps the inputs to a latent variable vector ***z***, and a generative model (the decoder) that decodes the latent variable vector back into the original data space. These two components mirror each other with a shared bottleneck layer, with the fewest nodes representing the latent generative factors. There could be several hidden middle layers between the bottleneck and the input layers. An illustration of a VAE model is shown in Figure 1.

The inference model estimates the posterior distribution of the latent factors in the bottleneck layer, which is assumed to be independent Gaussian. Consequently, the bottleneck layer consists of a vector of the means $\mu ={\left[\mu_{z1},\mu_{z2},\cdots \mu_{zd}\right]}^{T}$ and a vector of the standard deviations $\sigma ={\left[\sigma_{z1},\sigma_{z2},\cdots \sigma_{zd}\right]}^{T}$ of the posterior distribution. Then, samples drawn from the posterior distribution $z_{n}={\left[z_{1},z_{2}\ldots ,z_{d}\;\right]}^{T}$ are passed to the generative model to reconstruct the original input data.

Input data ${\;x}_{n}={\left[x_{1n},\;x_{2n,\;}\cdots x_{mn},\cdots x_{Mn}\right]}^{T}$ represent an *M* × 1 vector of personality variable scores from sample *n*, and *x*_*mn*_ denotes the *m*th personality variable. The **x**_*n*_ is assumed to follow a multivariate independent Gaussian distribution, and its mean and variances are modeled as a function of an *d*-dimension latent representation of personality traits ${\text{z}}_{n}\in {\mathbb{R}}^{d}$ by an encoder neural network *D*_θ_, where ***θ*** is a vector of encoder weights. Then the likelihood function of the input variable **x**_*n*_ can be defined as


(1) $$\begin{array}{l} p\left({\text{x}}_{n}|{\text{z}}_{n},\Theta \right)=p\left({\text{x}}_{n}|{\text{z}}_{n},\theta \right)\;=\;N\left({\mu_{\text{x}}}_{n},diag\left(\;\sigma_{x_{n}}^{2}\right)\right), \end{array}$$

where Θ = {θ,ϕ} is the combined vector of encoder and decoder weights. Since **x**_*n*_ only depends on the encoder weights, the model in (1) omitted decoder weights in the list of dependent variables. In VAE, **z**_*n*_ is commonly assumed to follow a prior distribution, which is the multivariate standard normal, i.e., $p\left({\text{z}}_{n}\right)=N\left(0,{\text{I}}_{d}\right)\;$ with **I**_**d**_ being a *d* × *d* identity matrix.

The goal of training or inference is to compute the maximum likelihood estimate of Θ


(2) $${\hat{\Theta }}_{ML}={\text{argmax}}_{\Theta }\;\sum_{n\;=\;1}^{N}\log p\left(x_{n}|\Theta \right)\approx {\text{argmax}}_{\Theta }\;\sum_{n=1}^{N}ℒ\left(\Theta \right)$$

where *p*(**x**_*n*_|**Θ**) = ∫*p*(**x**_*n*_|**z**_*n*_, **Θ**)*p*(**z**_*n*_)*d***z**_*n*_ is the marginal likelihood which is analytically intractable but can be lower bounded by the evidence lower bound (ELBO) *L*(Θ),


(3) $$ℒ\left(\Theta \right)=E_{q\left(z_{n}|x_{n},\Theta \right)}\left[\log p\left(x_{n}|z_{n},\Theta \right)\right]-D_{KL}[q\left(z_{n}|x_{n},\Theta \right)∥p(z_{n})]$$

where *q*(**z**_*n*_|**x**_*n*_, **Θ**) is an approximate to the intractable posterior distribution *p*(**z**_*n*_|**x**_*n*_, **Θ**), and *D*_*KL*_[*q*(**z**_*n*_|**x**_*n*_, **Θ**) ||*p*(**z**_*n*_)] measures the Kullback-Leibler distance (Walters-Williams and Li, 2010) between the approximated posterior distribution *q*(**z**_*n*_|**x**_*n*_, **Θ**) and the prior distribution *p*(**z**_*n*_). To make the variational inference tractable, *q*(**z**_*n*_|**x**_*n*_, **Θ**) is assumed in most cases as a multivariate Gaussian,


(4) $$\begin{array}{l} q\left({\text{z}}_{n}|{\text{x}}_{n},\Theta \right)=\;N\left({\mu_{z}}_{n},diag\left(\;\sigma_{z_{n}}^{2}\right)\right) \end{array}$$

whose means and variances $z_{n}={\left[z_{1},z_{2},\cdots z_{d}\right]}^{T}$ are given by a decoder network *E*_ϕ_ applied to *x*_*n*_ as


(5) $$\begin{array}{l} \left\{{\mu_{z}}_{n},\sigma_{z_{n}}^{2}\right\}=E_{\phi }\left({\text{x}}_{n}\right) \end{array}$$

where ϕ is the vector of the unknown decoder weights. Because of the approximation by L(Θ) in Equation (2) and the introduction of the decoder network in Equation (4), the model parameters to be estimated become Θ = {θ,ϕ}. Optimization of L(Θ) in Equation (2) is computed by the stochastic gradient descent algorithm, where the gradient is calculated by backpropagation. Note that the first part in Equation (3) will be proportional to the mean square error (MSE) between the input **x**_*n*_ and the reconstructed scores **v**_*x_n_*_ when we assume that the likelihood function of the personality variables follows an independent Gaussian distribution as in Equation (1). In VAE, the missing values are excluded when calculating the MSE.

VAE training is carried out with the TensorFlow machine learning module imported to Python (Jason, 2016).

### Model Accuracy Metric and VAE Model Selection

We calculate the Person Correlation between the input personality variables and the reconstructed ones (input-reconstruction correlation) as the performance metric. The R2 statistics can be calculated sample-wise, i.e., between **x**_*n*_ = [*x*_1*n*_, *x*_2*n*_, ⋯*x*_*Mn*_] and **v**_*n*_ = [*v*_1*n*_, *v*_2*n*_, ⋯*v*_*Mn*_] *n* ∈ (1, *N*), or variable-wise, i.e. between ${\text{x}}_{m}\;={\left[x_{m1\;,\;}x_{m2,\;}\cdots x_{mN\;}\right]}^{T}\;and\;{\text{v}}_{m}\;={\left[v_{m1\;,\;}v_{m2,\;}\cdots v_{mN\;}\right]}^{T}$ for all variable indices *m* ∈ (1, *M*). Note that the reconstructed variables are assumed to be the sum of input variables plus independent noise after the input is put through an encoder and decoder function. Other commonly used performance measures in LFA, such as the communality or the percentage of variance represented by the selected factors, are not applicable in the context of VAE because these measures assume a linear data model between the factors and the input data, while the encoder and decoder in VAE are not linear.

In the context of LFA, to calculate the Person Correlation between the input and the reconstructed personality variable scores, we can first reconstruct the score for the *m*th personality variable in the *n*th sample, *x*_*mn*_ based on the data model in LFA, which is a linear combination of *d* factors ${\text{z}}_{\text{n}}={\left[z_{1n},z_{2n}\ldots ,z_{dn}\;\right]}^{T}$ of the *n*th sample:


(6) $$\begin{array}{l} x_{mn}\;=\;l_{m1}z_{1n}\;+\;l_{m2}z_{2n}\cdots l_{md}z_{dn}\;+\;\varepsilon_{m}\;+\;\sigma_{mn} \end{array}$$

where ${\text{l}}_{m}={\left[l_{m1},l_{m2}\ldots ,l_{md}\;\right]}^{T}\;$ is the vector of factor loadings in the *m*th personality variable, ε_*m*_ is the item specific factor, and σ_*mn*_ is the observation noise for the *m*th personality variable in the *n*th sample. Then the reconstructed score becomes *v*_*mn*_ = ${{\text{l}}_{m}}^{T}{\text{z}}_{\text{n}}$ if we assume that both ε_*m*_ *and σ*_*mn*_ have zero means. Then the correlation between the input and the reconstructed personality variables can be calculated either sample-wise or variable-wise for LFA.

The *d* latent factors ${\text{z}}_{\text{n}}={\left[z_{1n},z_{2n}\ldots ,z_{dn}\;\right]}^{T}$ are calculated both by the Thurstone and the Bartlett (Grice, 2001) methods. We compared the results of reconstruction and found that the two methods did not make a significant difference on input-reconstruction correlations in the tested datasets.

Note that in the machine learning community, the commonly used measure is Coefficient of Determinate (R2 statistics), which is defined as one minus the total error variance divided by the total sample variance. In the context of VAE, it can be viewed like communality in LFA, which measures how much variance has been explained.

In VAE, the *m*th personality variable's loadings on the *i*th factor, *l*_*mi*_, is estimated by calculating the correlation between the latent factor's mean vector $\mu_{zi}\;={\left[\mu_{zi1},\mu_{zi2},\cdots \mu_{ziN}\right]}^{T}$, with the *m*th reconstructed input personality variable ${\text{v}}_{m}\;={\left[v_{m1\;,\;}v_{m2,\;}\cdots v_{mN\;}\right]}^{T}$ over all *N* training samples. Note that this loading is calculated for the purpose of finding a rotation of the factors such that the latent factors from different VAE runs can be aligned. We used the reconstructed inputs for loading calculation because the effect of noise has been removed after the reconstruction process and **μ**_**zi**_ represents the maximum posterior (MAP) estimation of the latent variables.

In LFA, the *m*th personality variable's loadings on the *i*th factor, *l*_*mi*_, is estimated by calculating the correlation between the vector of latent factor $z_{i}={\left[z_{i1\;}z_{i2,\;}\cdots z_{iN\;}\right]}^{T}$ with the vector of the *m*th input personality variable ${\text{x}}_{m}\;={\left[x_{m1\;,\;}x_{m2,\;}\cdots x_{mN\;}\right]}^{T}$ over all *N* training samples. The calculation is performed by the factor analyzer in Python. Since we do not compare VAE and LFA in factor loadings, the difference in the calculation procedure of these loading factors is not consequential for the interpretation of the results.

### VAE Factor Stability Analysis Based on Congruence Scores Over Multiple Runs

For LFA, noise factors beyond the first 5 did not appear consistently in different studies (Goldberg, 1990). Factors from different LFA runs could be rotated or perturbed due to variations, which can be attributed either to the analyzing methods or the datasets. Similarly, we also exclude noise factors and determine stable factors to be included in VAE constructed models.

The VAE employs a stochastic gradient descent algorithm that may not converge to the same solution across multiple runs. Such variations may introduce noise factors in addition to the ones introduced by the variations in the training datasets. To make sure that we only include stable factors in the final personality model, we extended the concept of congruence coefficient introduced by Goldberg for studying factor stability across different methods (Goldberg, 1990) and defined the congruence score between any two factors *i* and *j* in run *r1* and run *r2* as: $C_{i,j}^{r1,r2}=Corr\left(l_{i}^{r1}{,l}_{j}^{r2}\right)$, where ${\text{l}}_{i}={\left[l_{1i},l_{2i}\ldots ,l_{Mi}\;\right]}^{T}$ represents the *i*th factor loadings on all M personality variables, and *Corr()* represents the Pearson correlation function.

In each VAE run, the latent factors are supposed to be independent and for each factor, only one factor from another run can be matched with it with a high congruence score. However, VAE often returns correlated factors within a run when we set the number of bottleneck layer nodes higher than the actual number of stable factors. In such cases, multiple factors from the same run may be clustered together with a given factor from another run. We observed that falsely matched factors generally have lower congruence scores than the true matching factors. To prevent the clustering algorithm from falsely matching factors, we set up a threshold and removed congruence scores below the threshold.

After the filtering step, the Leiden clustering algorithm (Traag et al., 2019) was applied to cluster factors from different runs. The Leiden clustering algorithm was developed to improve the Louvain algorithm. The Leiden algorithm allows both splitting and merging. The Leiden algorithm guarantees that clusters are well-connected and the clusters it finds are not too far from optimal.

All matching factors from all runs will be reported as clusters by the Leiden clustering algorithm. Then, we manually validated the clusters returned by the Leiden algorithm by inspecting the personality variables associated with each factor. To ensure that factors from different runs can be aligned together, we performed varimax rotation on the factor loadings before they were used to calculate the congruence scores.

To apply the clustering algorithm, d factor loading vectors from all R runs are retrieved for calculating a congruence score/factor loading correlation matrix with (dR) ^*^ (dR) elements. Then, the elements in the congruence score matrix below the threshold will be set to zero before it is fed into the Leiden clustering algorithms.

In practice, the threshold on the congruence scores was gradually raised until factors from the same run cannot be clustered together. We define a factor as stable when it can be identified in each VAE run. The average of the congruence scores within a cluster is used to represent the overall factor congruence.

We determine the final number of stable factors by increasing the number of bottleneck layer nodes until no more stable factors emerge.

### Determining Factor-Variable Associations in VAE

After VAE identified a set of latent factors, it is important to understand how these factors can be interpreted. In LFA, this is done by inspecting the factor loadings of personality variables. However, since factor loadings are calculated by assuming a linear relationship between the factors and personality variables, we cannot apply the same method for identifying factor-variable association in VAE. We propose to inspect the reduction of correlations between the input and reconstructed personality variables. To calculate the reduction, we first set the investigated latent factor to zero, while the rest of the factors are fed into the VAE decoder to reconstruct the personality variables across all samples. Then, we calculate the correlations between the input and reconstructed variables after muting the investigated factor and compare it to the correlations before muting the factor. Personality variables with input-reconstruction correlation reduction above a threshold are associated with a latent factor. Since this method does not make any assumption on the linearity of the encoding and the decoding process, it is suitable for inspecting factor-variable association in both VAE and LFA.

### Cross-Regional Study

To see whether the VAE returned factor model is valid across different regions, we trained a VAE model based on 80% of the samples from North America in the HEXACO analysis. Then, we estimated the factor statistics, especially the mean, the standard deviation, and the zero-order correlations of the derived factors to see if the factor model varies across different regions.

### Linear Factor Analysis (LFA)

The LFA was conducted using the python function FactorAnalyzer imported from the sklearn.decomposition module (Persson and Khojasteh, 2021). Since previous research indicated that the selection of the LFA method would not make a significant difference in LFA (Goldberg, 1990), we used the principal factor method included in the package and selected ‘varimax' as the rotation method. The same scaled and normalized input data were used for both LFA and VAE.

### Testing and Training Dataset Separation

We separated each input dataset into a training and a testing dataset by an 80–20% ratio. The training dataset was used for training the weights in the encoder and the decoder in VAE. It was also used to train the factor analyzer in LFA, which generated the factor loading matrix, a factor transformer (by default the Thurstone method) that can be used to calculate the factor scores, and other statistics, such as the eigenvalues of the data. We have also trained the weights for calculating the factor scores in the Bartlett method.

In VAE, the testing dataset was used to estimate the latent factors using the decoder, the reconstructed personality variables by using the encoder and the decoder, the input-reconstruction correlations, the congruence scores in factor stability analysis, and input-reconstruction correlation reduction in factor-variable association analysis. In LFA, the testing dataset was used to calculate the factor scores, the reconstructed personality variables, and the rest of the measures as in VAE.

In VAE training, the training dataset was further split into a training and validation dataset to ensure that the algorithm does not over-fit. The split ratio is 80–20%.

Complete separation between the training and the testing datasets is ensured. This procedure reduces the risk of overfitting because all testing was conducted on samples not included in the model construction process. Ten VAE models were trained using 10 training datasets in all analyses.

## Results

### Analysis of the IPIP Big 5 Dataset

#### LFA Exploratory Factor Analysis

**Figure 2:**
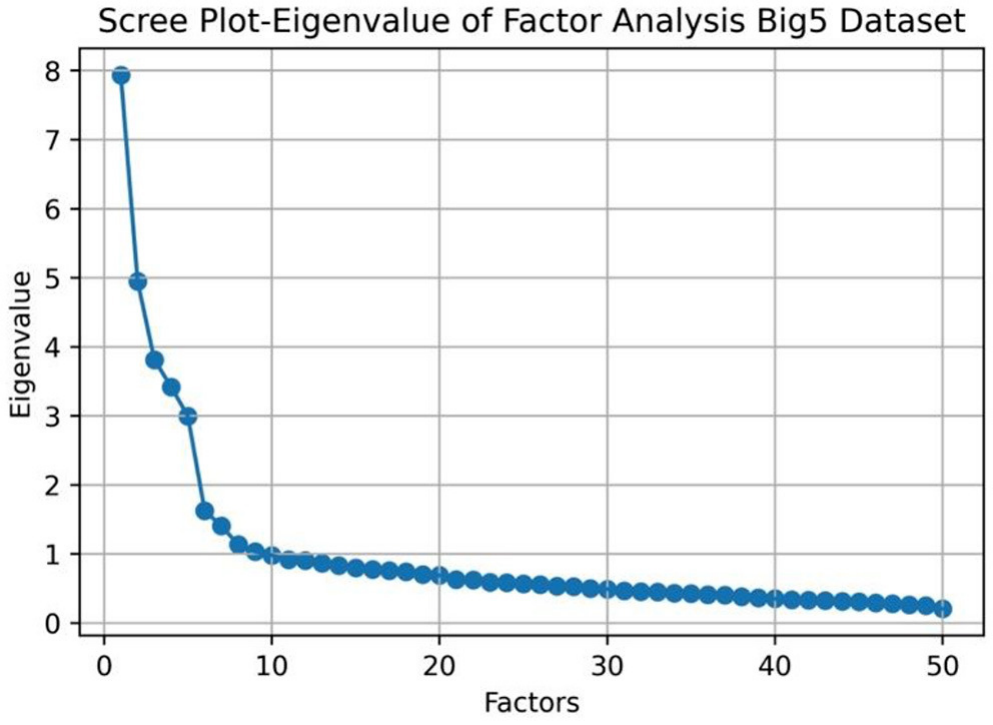
Exploratory factor analysis of the IPIP Big 5 dataset.

We first conducted a LFA exploratory analysis by plotting the eigenvalues of the dataset. The scree plot in Figure 2 shows that there are 7 factors with eigenvalues greater than 1. If we look for the factors left to the elbow point, there are 5 factors in the data. Past research found that there are 5 stable factors (Costa and McCrae, 1992; Goldberg, 1992) that emerge from run to run. We can see that the results from the eigenvalue analysis and the scree plot are different and fewer factors can be generalized.

**Figure 3:**
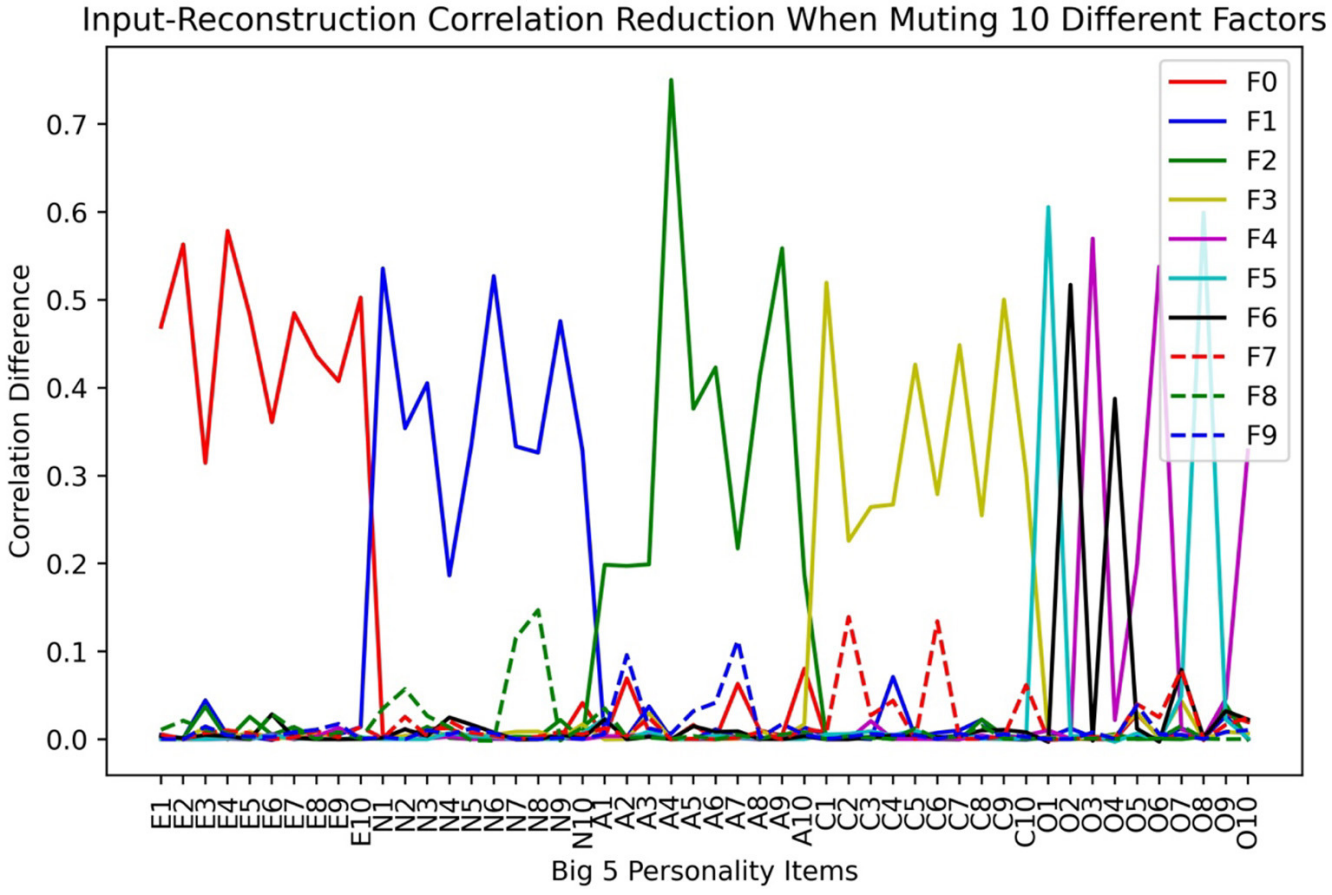
Input-reconstruction correlation reduction when assuming 10 Latent Factors in LFA.

We then analyzed the factor-variable associations by inspecting the input-reconstruction correlation reduction as we increased the number of factors in LFA. An example result is shown in Figure 3 when there are 10 factors. We can see that the first 4 four factors of Extroversion, Neuroticism, Agreeableness, and Conscientiousness stayed the same as the original scale. However, the Openness factor fractionated in to 3 smaller factors, which makes the total number of factors to be 7.

#### VAE Analysis of the IPIP Big 5 Dataset

##### Model Parameter Selection

Model accuracy is measured by the variable-wise input-reconstruction correlations. Table 1 shows the results when using different numbers of hidden middle layers, different numbers of hidden middle layer nodes, and different numbers of bottleneck layer nodes. Note that the activation function of the last layer of the generative model is linear so that the output can have negative results. The activation function of bottleneck layer nodes must also be linear because they are assumed to be Gaussian variables. The Relu activation function was used for the rest of the layers.

**Table 1:** The Mean (Std) of variable-wise input-reconstruction correlations.

	**# Bottleneck nodes-1 layer**		**# Bottleneck nodes-two layers**	
**# Mid-Layer Nodes**	**8**	**10**	**8**	**10**
50	0.694 (0.066)	0.714 (0.062)	0.670 (0.076)	0.671 (0.075)
100	0.728 (0.061)	0.748 (0.057)	0.715 (0.058)	0.716 (0.059)
200	0.731 (0.058)	0.752 (0.050)	0.730 (0.057)	0.750 (0.048)

*N = 3,944 samples from the testing dataset. When two middle layers are used, the number of nodes used in the second mid-layer is half of that in the first middle layer*.

Inspecting how the mean of the input-reconstruction correlations changes as we increase the number of middle layer nodes from 50 to 200 in Table 1, we can see that increasing the number of middle layer nodes constantly improves the performance, although increasing it beyond 100 nodes offered little performance gain. Further increasing the number of nodes does not change the factor structure or improve the correlations between the input and reconstructed variables significantly. Meanwhile the computational cost will grow significantly. Also, as the model becomes more complex, the required number of samples for appropriately estimating the weights in the deep neural network will increase. Another point is that in Table 1, it is evident that using two middle layers did not improve the performance. These observations hold with different the number of bottleneck layer nodes. Consequently, it should be sufficient to double the number of input layer nodes for the single middle layer.

**Figure 4:**
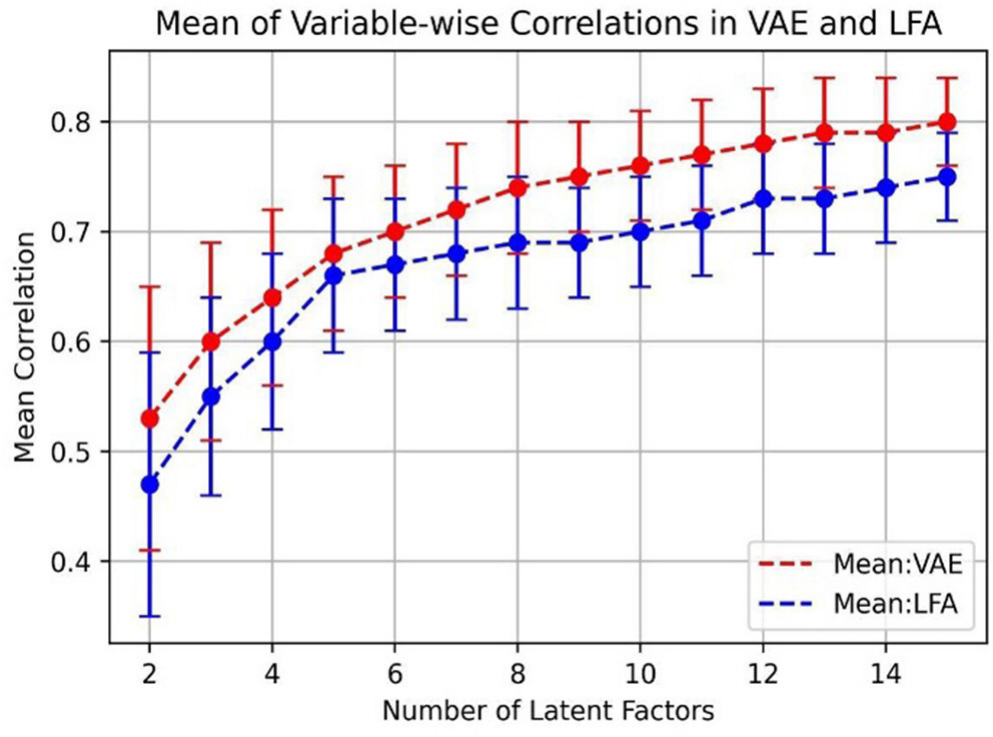
Mean of input-reconstruction correlations in VAE and LFA.

To further investigate the impact of the number of bottleneck layer nodes, in Figure 4, we plot the means and the standard deviations of variable-wise input-reconstruction correlations. The number of latent factors was increased from 2 to 15. The standard deviations are indicated by the error bars in the plot. These results are evaluated based on the testing dataset with 3,944 samples.

The improvement on the mean of correlations significantly slows down after the 5th latent factor in LFA, consistent with previous results (Costa and McCrae, 1992; Goldberg, 1992). On the other hand, the performance of VAE keeps on improving as the number of bottleneck layer nodes increases and there is not an obvious “elbow” point as in LFA for determining the number of factors to be extracted.

**Figure 5:**
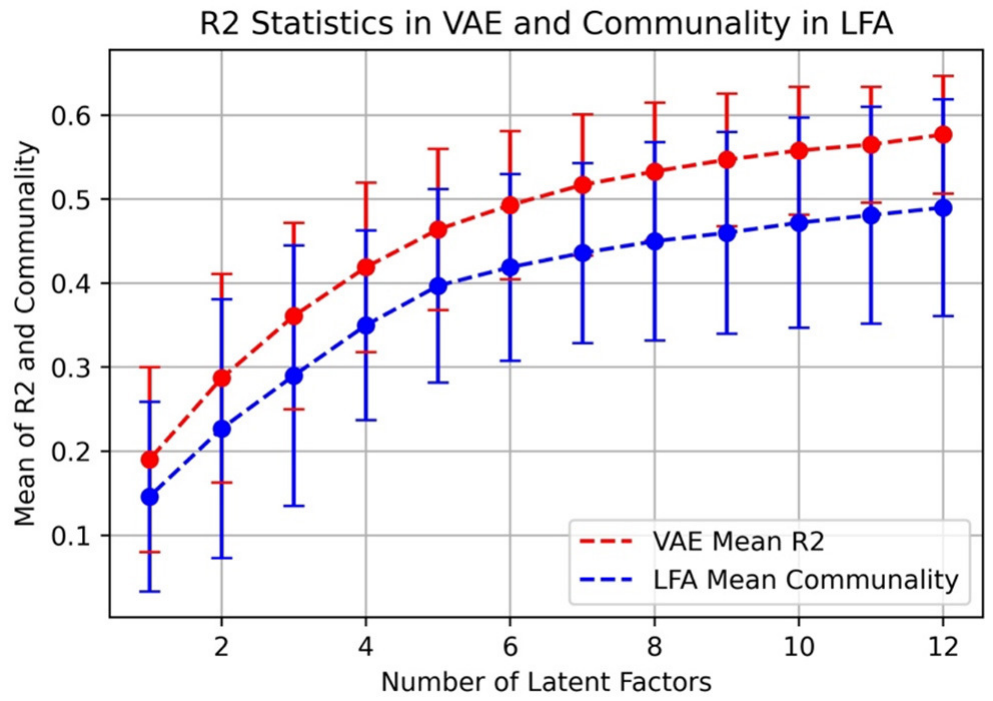
Mean of R2 statistics in VAE and communality in LFA.

In Figure 5, we also compared R2 statistics in VAE to communality in LFA because it measures explained variance.

We can see that in Figure 5, R2 statistics in VAE outperforms communality in LFA and the curves reflect the same trend as in Figure 4. Note that if the linear model in LFA completely holds, then communality should be the equivalent to R2. To plot (Figure 5), we first calculated the two measures (R2 and communality) for each variable, and then, we calculated the mean and standard deviation of the two measures across all 50 IPIP Big5 variables.

##### Factor Stability Analysis

**Figure 6:**
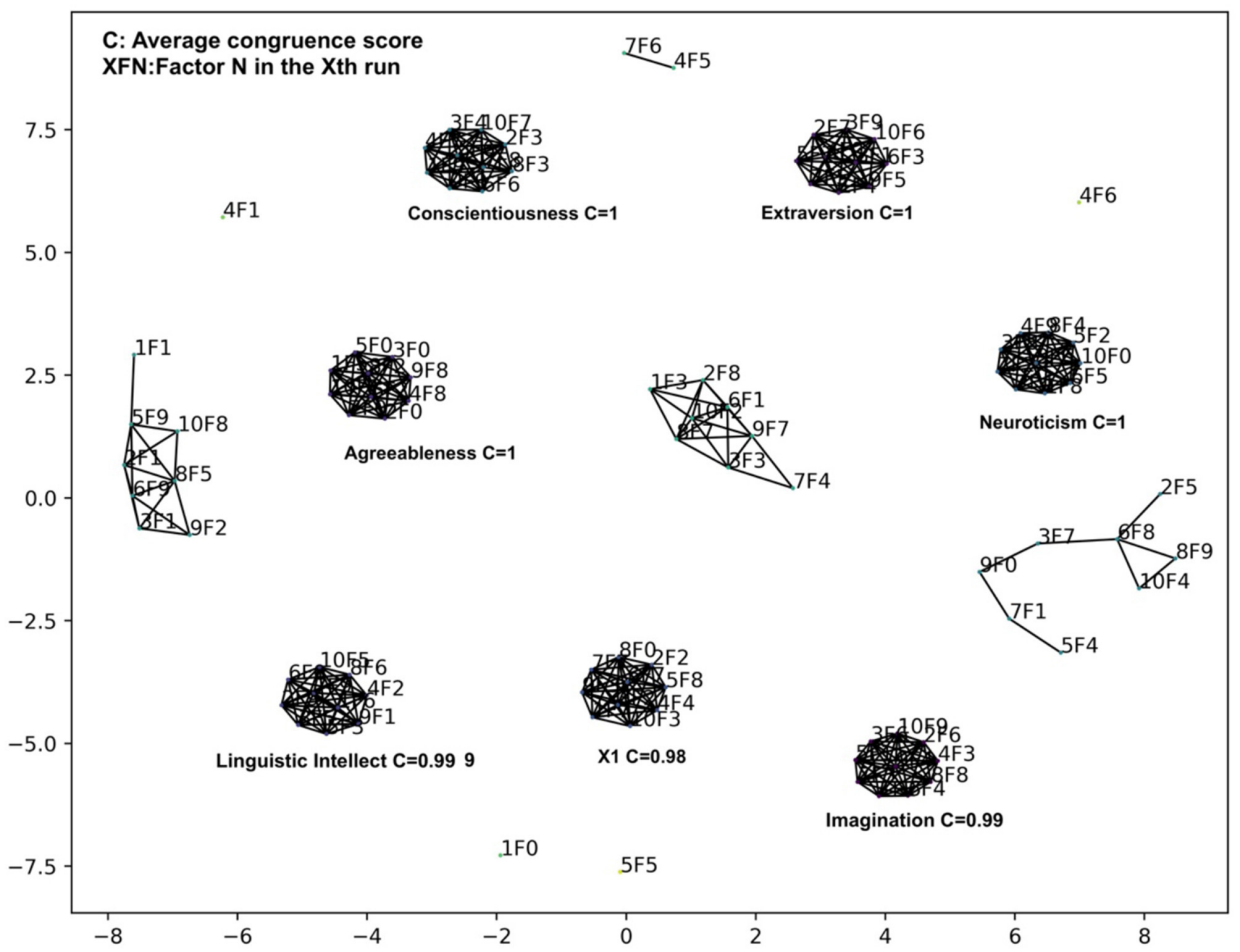
Clustering of factors from 10 VAE runs with 10 bottleneck layer nodes and 100 middle layer nodes in the Big 5 analysis.

From the plot in Figure 5, we cannot determine the number of factors that should be included in the personality model, and we further studied the stability of the discovered factors following the procedure outlined in the method section and summarized the results in Figure 6, in which we used 10 bottleneck layer nodes and 100 middle layer nodes.

After varimax rotation, we gradually raised the threshold on the congruence scores until no two factors from the same run could be clustered together. The threshold was set to 0.90. We can see that 7 stable factor clusters emerged with average congruence scores greater than 0.98. For the first 4 factors, Extraversion, Agreeableness, Conscientiousness, and Neuroticism, the top 10 personality variables ranked by factor loadings are the same as those in the original Big 5 model. The average congruence scores of these factor clusters are very high, which are rounded to one when two decimal points are considered. The rest of the 3 factors have a slightly smaller congruence score. Since factor loadings are not very appropriate for exploring factor-variable association in VAE and these factors have relatively less obvious interpretations, we temporarily mark them as Imagination, Linguistic Intellect, and a factor X1. The X1 did not have high loadings on any items and so we did not assign a name to it.

We also investigated the case of using 9 bottleneck layer nodes and 100 middle hidden layer nodes. The stability analysis results are included in Supplementary Figure 1.

##### Factor-Variable Associations

**Figure 7:**
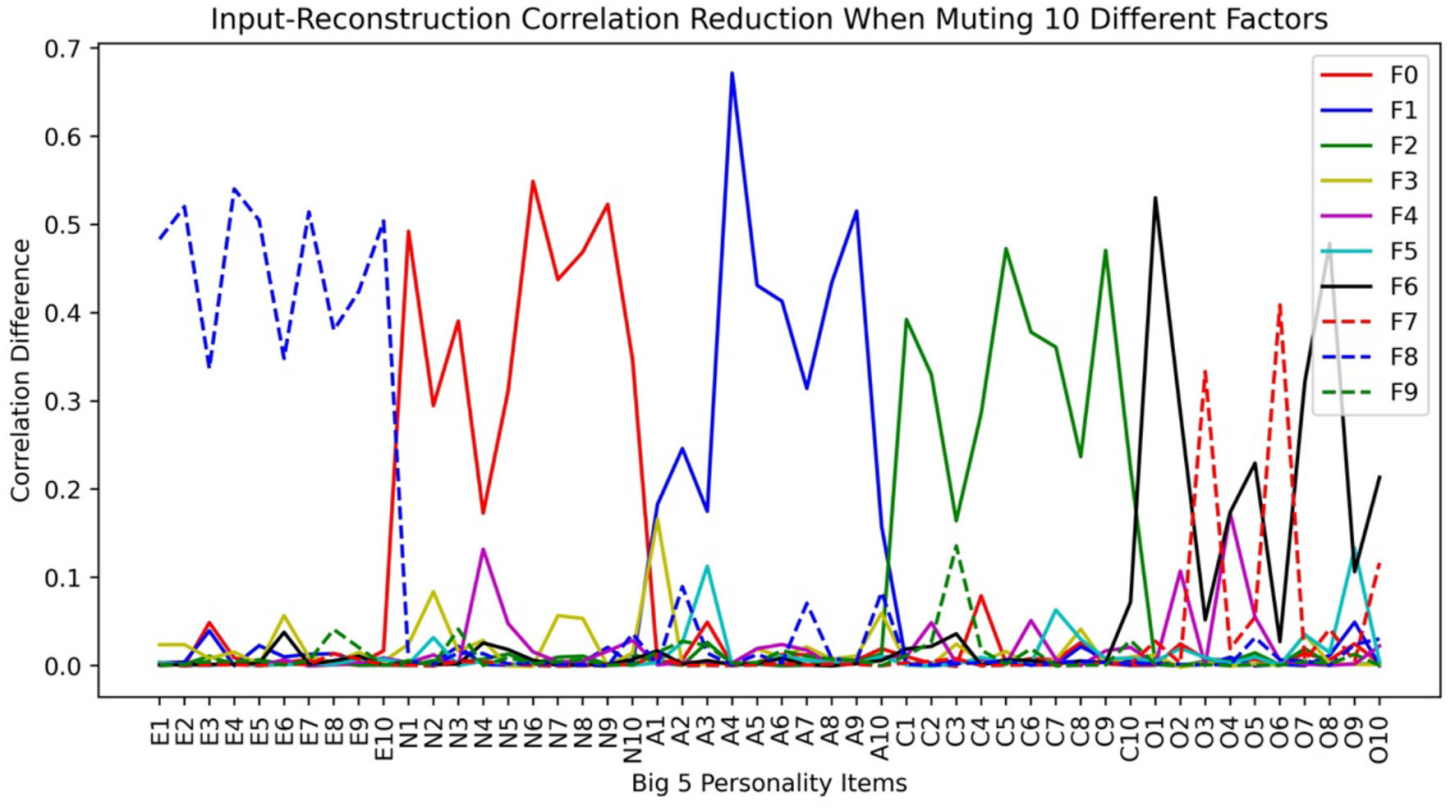
Input-reconstruction correlation reduction in a Big5 VAE run (E, Extraversion; N, Neuroticism; A, Agreeableness; C, Conscientiousness; O, Openness. See Table 2 for variable definitions).

In Figure 7, we plot the input-reconstruction correlation reduction after muting each factor when 10 bottleneck layer nodes are used.

**Table 2:** Factor-variable association by inspecting input-reconstruction correlation reduction with 10 bottleneck layer nodes.

	**Reduction**	**Item**
Factor	Neuroticism	
N6	0.54880679	I get upset easily.
N9	0.52292319	I get irritated easily.
N1	0.49218794	I get stressed out easily.
N8	0.46884062	I have frequent mood swings.
N7	0.43760175	I change my mood a lot.
N3	0.39057886	I worry about things.
N10	0.34668783	I often feel blue.
N5	0.3111992	I am easily disturbed.
N2	0.29470729	I am relaxed most of the time.
N4	0.17285815	I seldom feel blue.
Factor	Agreeableness	
A4	0.67151324	I sympathize with others' feelings.
A9	0.51527633	I feel others' emotions.
A8	0.43419151	I take time out for others.
A5	0.43105851	I am not interested in other people's problems.
A6	0.41301325	I have a soft heart.
A7	0.31421132	I am not really interested in others.
A2	0.24600361	I am interested in people.
A1	0.18277531	I feel little concern for others.
A3	0.1748963	I insult people.
A10	0.15831119	I make people feel at ease.
Factor	Conscientiousness	
C5	0.4724694	I get chores done right away.
C9	0.47059017	I follow a schedule.
C1	0.39228489	I am always prepared.
C6	0.37797469	I often forget to put things back in their proper place.
C7	0.361025	I like order.
C2	0.32991215	I leave my belongings around.
C4	0.28578476	I make a mess of things.
C8	0.23683019	I shirk my duties.
C10	0.22386922	I am exacting in my work.
C3	0.16418281	I pay attention to details.
Factor	X1	
A1	0.16598865	I feel little concern for others.
Factor	Not Stable	
O4	0.17283142	I am not interested in abstract ideas.
N4	0.13201049	I seldom feel blue.
O2	0.10712201	I have difficulty understanding abstract ideas.
Factor	Not Stable	
O9	0.13302234	I spend time reflecting on things.
A3	0.11271361	I insult people.
Factor	Linguistic Intellect	
O1	0.53025199	I have a rich vocabulary.
O8	0.47849211	I use difficult words.
O7	0.32149661	I am quick to understand things.
O2	0.28731204	I have difficulty understanding abstract ideas.
O5	0.2294474	I have excellent ideas.
O10	0.21341601	I am full of ideas.
O4	0.17363996	I am not interested in abstract ideas.
O9	0.10652723	I spend time reflecting on things.
Factor	Imagination	
O6	0.40875143	I do not have a good imagination.
O3	0.33323853	I have a vivid imagination.
O10	0.1164824	I am full of ideas.
Factor	Extraversion	
E4	0.54024501	I keep in the background.
E2	0.52025504	I don't talk a lot.
E7	0.51419152	I talk to a lot of different people at parties.
E5	0.50472147	I start conversations.
E10	0.50394706	I am quiet around strangers.
E1	0.4833213	I am the life of the party.
E9	0.42410076	I don't mind being the center of attention.
E8	0.38028153	I don't like to draw attention to myself.
E6	0.34816174	I have little to say.
E3	0.33659072	I feel comfortable around people.
Factor	Not stable	
C3	0.13559234	I pay attention to details.

*N = 3,944 testing samples; Only variables with correlation reduction higher than 0.1 are kept for each factor*.

From Figure 7, we can see that the factor-variable associations are almost the same as in the LFA analysis. The openness factor fractionated into Imagination (F7 in Figure 7), Linguistic Intellect (F6), and X1 (F3). X1 is a minor factor in the sense that its main items have small reductions. However, it withstood the test of stability, and it cannot be ignored as noise. Compared to Figure 3, we can see that while LFA fractionated Openness into 3 factors, VAE fractionated it into two. LFA tends to fractionate more as we increase the number of assumed latent factors.

Inspecting the variables associated with each factor in Table 2, we can see that by using input-reconstruction correlation reduction, it is possible to identify factor-variable associations that mostly reproduce the results in the Big5 model.

We listed the correlations between the 7 factors in Table 3.

**Table 3:** Correlations between the 7 stable factors in the VAE IPIP Big 5 dataset analysis.

	**(1)**	**(2)**	**(3)**	**(4)**	**(5)**	**(6)**
(1) Conscientiousness	-					
(2) Linguistic Intellect	−0.03	-				
(3) Extraversion	−0.05^*^	−0.02	-			
(4) Agreeableness	0.08^**^	−0.01	0.07^**^	-		
(5) X1	0.01	−0.05^*^	0.02	0	-	
(6) Neuroticism	0	0.03	−0.05^*^	0.06^**^	−0.03	-
(7) Imagination	0.02	0.1^**^	−0.02	−0.03	0.08^**^	−0.02

*N = 3,944*.

* *P < 0.05; Bold values and*

** *are indicate that P-value < 0.001*.

**Figure 8:**
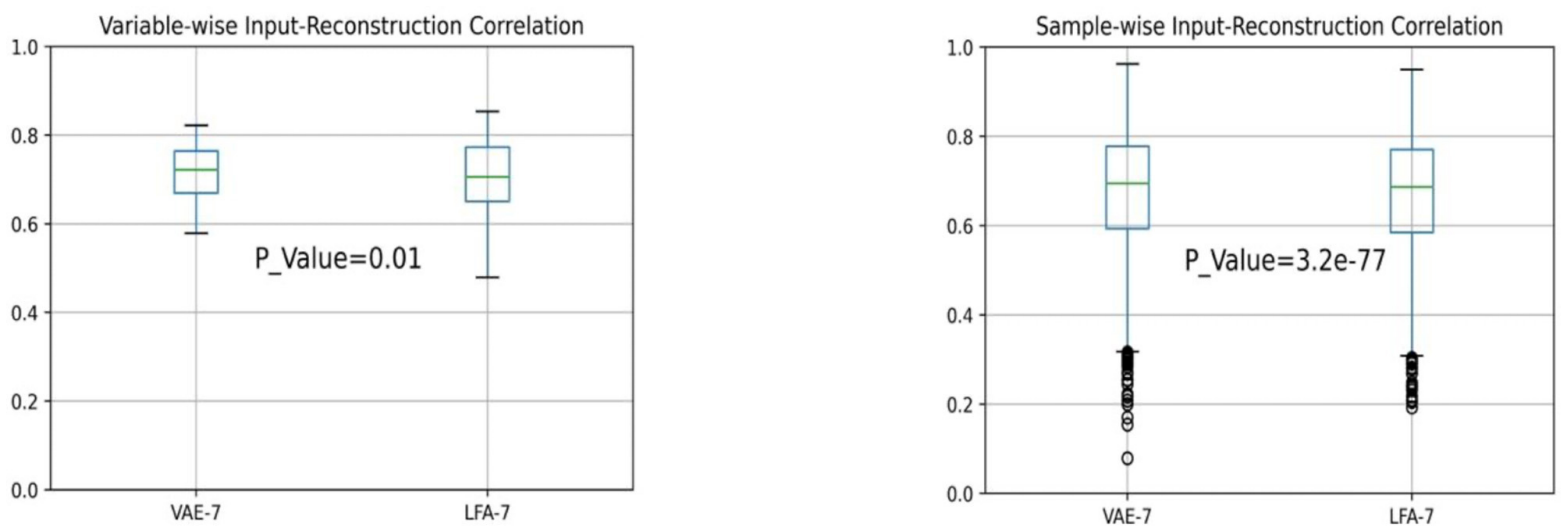
Variable-wise and sample-wise correlation statistics in VAE and LFA.

We have also calculated the sample-wise and variable-wise correlations between the reconstructed and the original inputs in both analyses over 3,944 testing samples and 50 personality variables. We used 7 hidden layer nodes for VAE and 7 factors for LFA for a fair comparison. We applied the Wilcoxon signed-rank test implemented in the SciPy package in python (Scipy Wilcoxon Signed-Rank Test Manual, n.d.) and calculated the *p*-values. The resulting statistics are shown in the box plots in Figure 8. They show that the 7-factor VAE model performs better than the 7-factor LFA model. The variance of variable-wise correlation is significantly smaller in VAE than LFA.

In this study, we can see that VAE mostly replicated the factor structure of the Big 5 scale initially discovered by LFA.

### Analysis of the IPIP HEXACO Dataset

In the VAE analysis of the IPIP Big 5 data, the inventory only contains 50 personality variables. We wanted to investigate if VAE can be used when more personality variables are on the questionnaire and to verify if the factor selection principle derived in the first VAE analysis can be generalized. For this purpose, we applied VAE to an IPIP HEXACO dataset with 240 personality variables. We selected the model structure according to the principles derived previously. Also, since the IPIP HEXACO dataset has many more items than the Big5 dataset, we anticipated more factors.

#### LFA Analysis of the IPIP HEXACO Dataset

**Figure 9:**
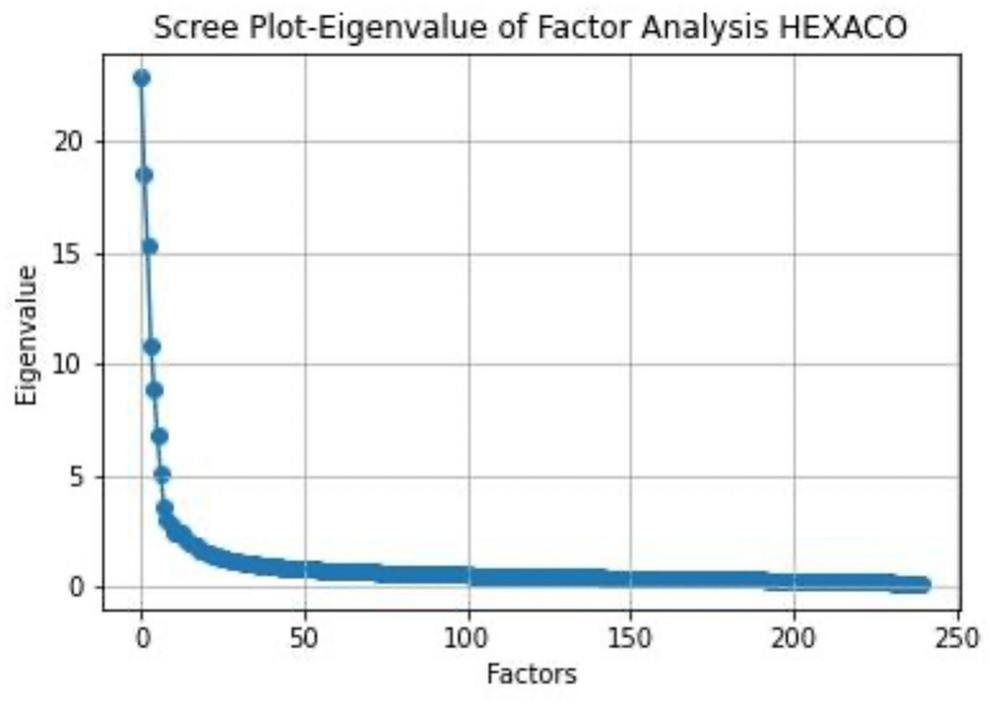
Scree plot of the eigenvalues in the HEXACO dataset.

We first applied LFA analysis of the dataset and the resulting eigenvalues are plotted in the following scree plot:

From Figure 9, we can see that there are about 8 factors before the elbow point. However, if we applied the rule of selecting factors with eigenvalues greater than one, there are over 37 factors with eigenvalues above 1. Yet in the literature, the reported number of factors through LFA analysis is 6.

**Figure 10:**
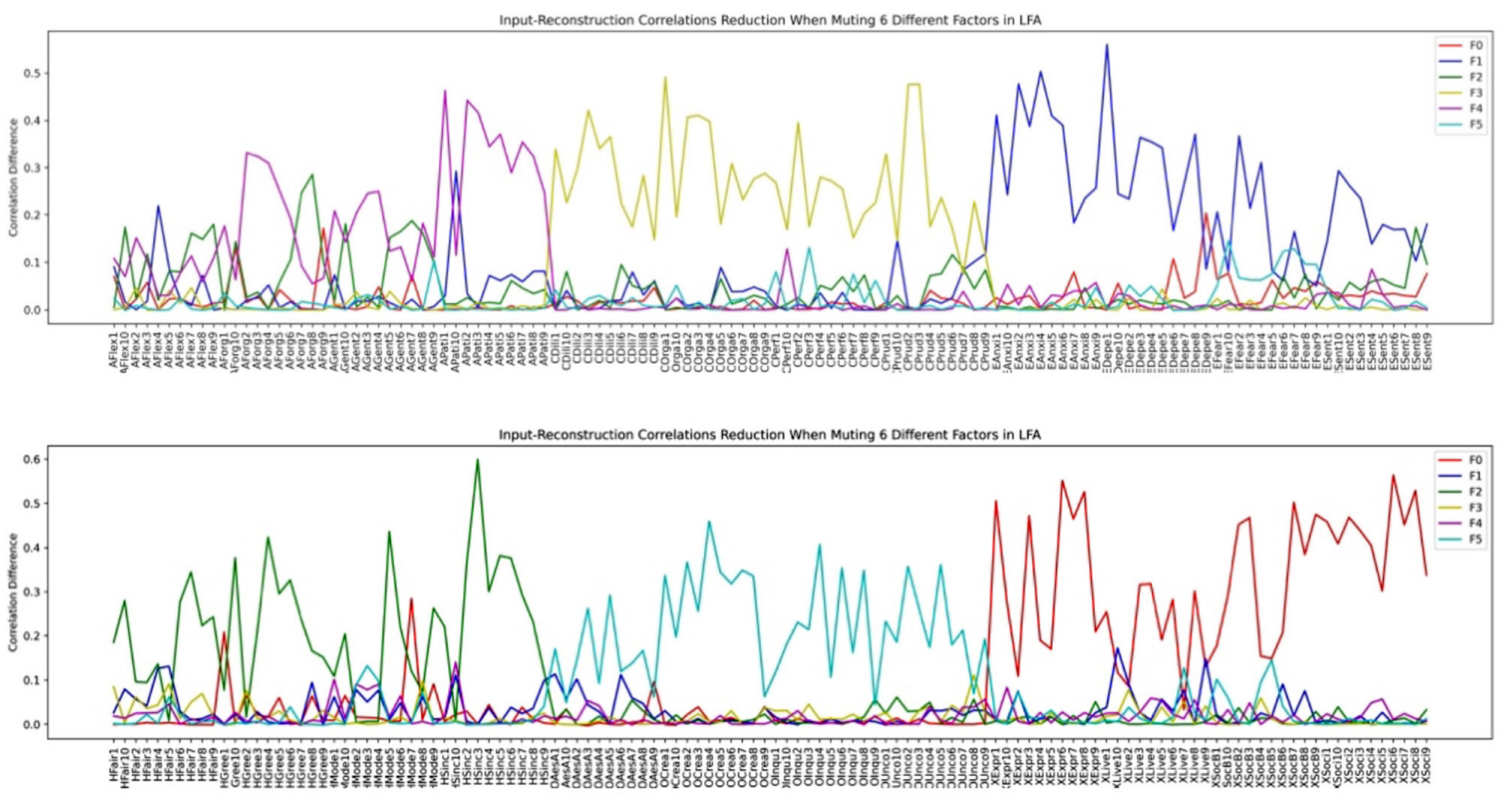
HEXACO factor- variable association when 6 latent factors are assumed in LFA (see section Methods for the list of acronyms).

Since there is a big discrepancy in the number of factors that should be extracted according to various factor extraction criteria, we investigated the factor structure when different numbers of latent factors are assumed in LFA. We first plotted the input-reconstruction correlation reduction plot when we assumed 6 latent factors and each of them was muted in turn in Figure 10. We can see that the plot reflects the standard HEXACO model structure because each factor is represented by a line with significant input-reconstruction correlation reduction over variables grouped for one factor and lower for other variables except some facets of Agreeableness and Humility-Honesty. Some of these facets are influenced by both the Agreeable and Humility-Honesty factor. We know that the factor level description is not sufficient to describe the complexity of the personality model. Personality variables associated with each factor are further divided into facets.

**Figure 11:**
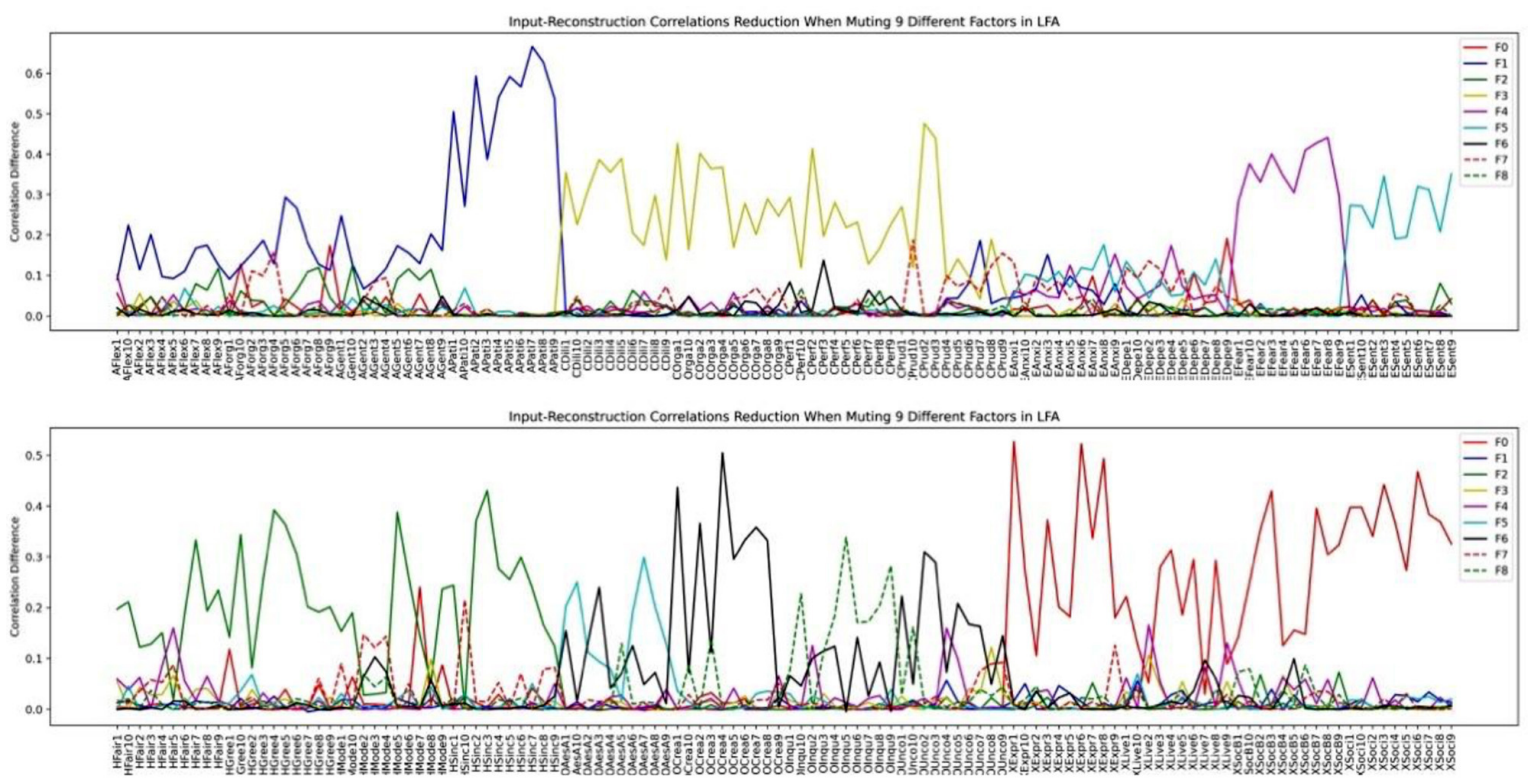
HEXACO factor-variable association when 9 latent factors are assumed in LFA.

**Figure 12:**
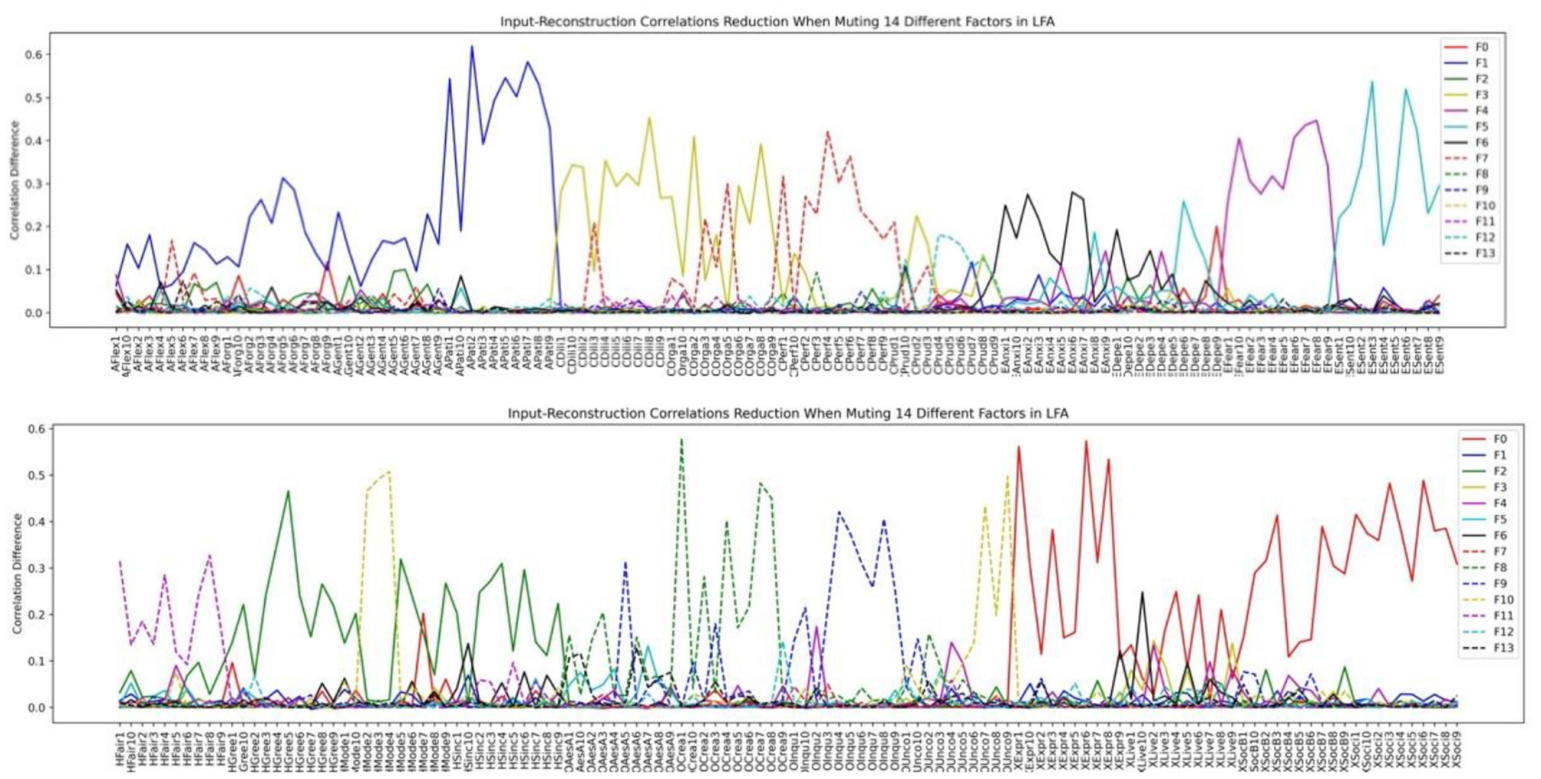
HEXACO factor-personality variable association when 14 latent factors are assumed in LFA.

We then investigated how the factor structure would change when we assume the number of latent factors to be 9 and 14 in Figures 11, 12, respectively.

Inspecting the factor structure when we assume 6, 9, and 14 factors, respectively, we can see that the factor structure keeps on fractionating without stability. The factor structure also does not improve the representation of the model as the number of factors increases. For example, the Depression and the Anxiety facet in Emotionality is well-represented in the 6-factor structure in Figure 10. However, when the number of factors is increased to 9, these two facets are influenced by several factors in which the factor plotted by the red dashed line (F7) is not representing any facet or factor (see the top half of Figure 11). In the case of 14 factors in Figure 12, we can see that except Extraversion and Agreeableness, all the rest of the factors are fractionated and there are 12 factors with a peak reduction greater than 0.2 and more than 5 variables. The structure is different from the 9-factor case, in which 9 factors have a peak reduction of greater than 0.2.

It is evident that when we apply LFA to factor analysis to a large set of personality variables, it is hard to determine the number of factors to be extracted based on eigenvalues or elbow points. The factor structure keeps on fractionating into smaller but still significant factors, and there is no definitive way to determine when to stop.

#### VAE Model Parameter Selection

We employed the model selection principles used in the VAE IPIP Big 5 dataset analysis. We set the number of nodes in the middle layer to 480, twice the number of input variables. Further increasing the number of nodes brought little improvement. A single middle layer between the input and the bottleneck layer was used and the activation function for the mid-layer was set to Relu. We gradually increased the number of bottleneck layer nodes to 12 and a maximum of 9 stable factors emerged in the analysis.

#### Factor Stability Analysis

**Figure 13:**
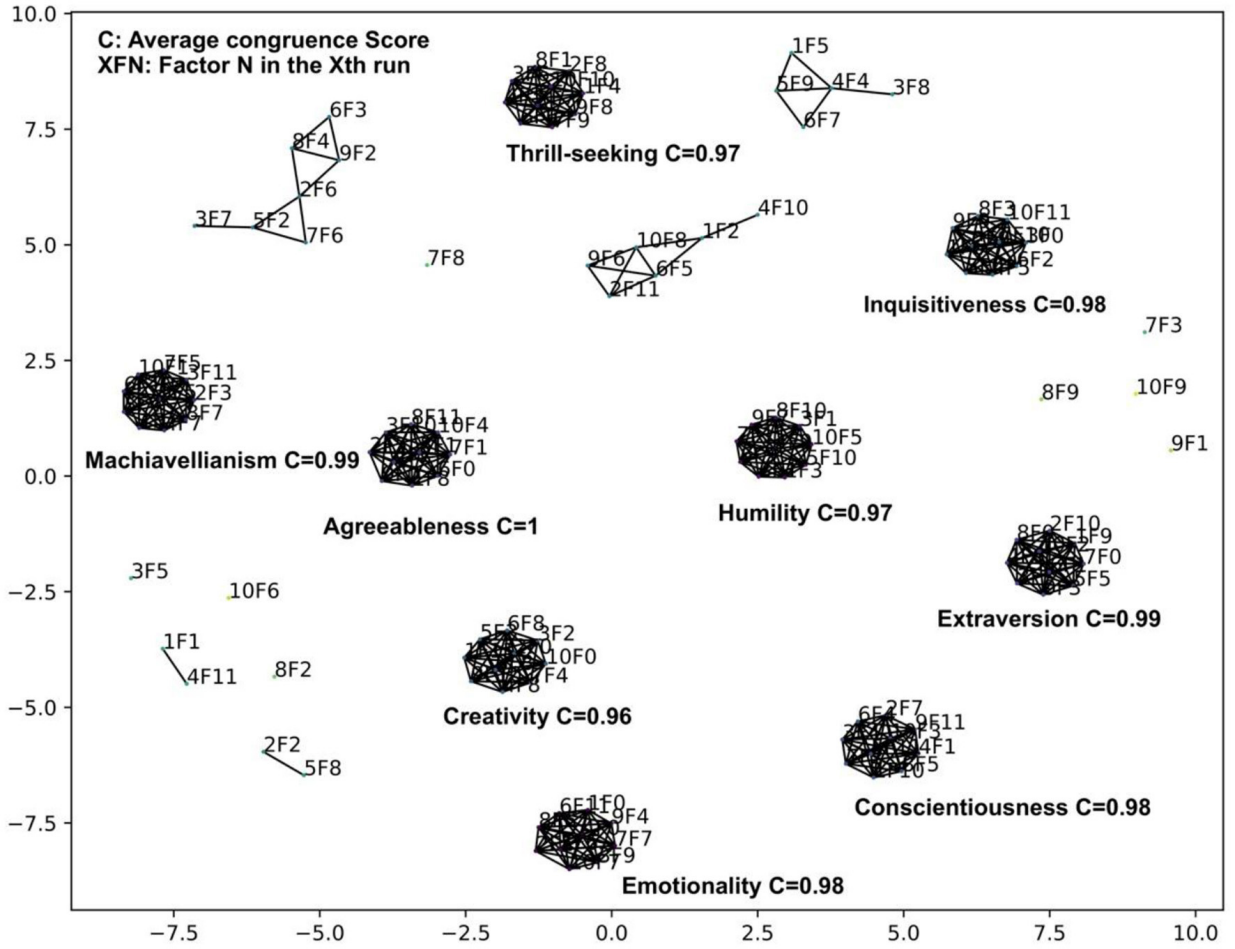
VAE analysis of the IPIP HEXACO dataset using 12 bottleneck layer nodes.

The result of factor stability analysis on the 240 IPIP HEXACO variables is shown in Figure 13.

The filter threshold on the congruence scores was set to 0.875 such that no single factor is matched to two factors in another run. Nine stable factors with an average congruence score greater than 0.97 appeared across 10 VAE runs with 12 bottleneck layer nodes.

**Figure 14:**
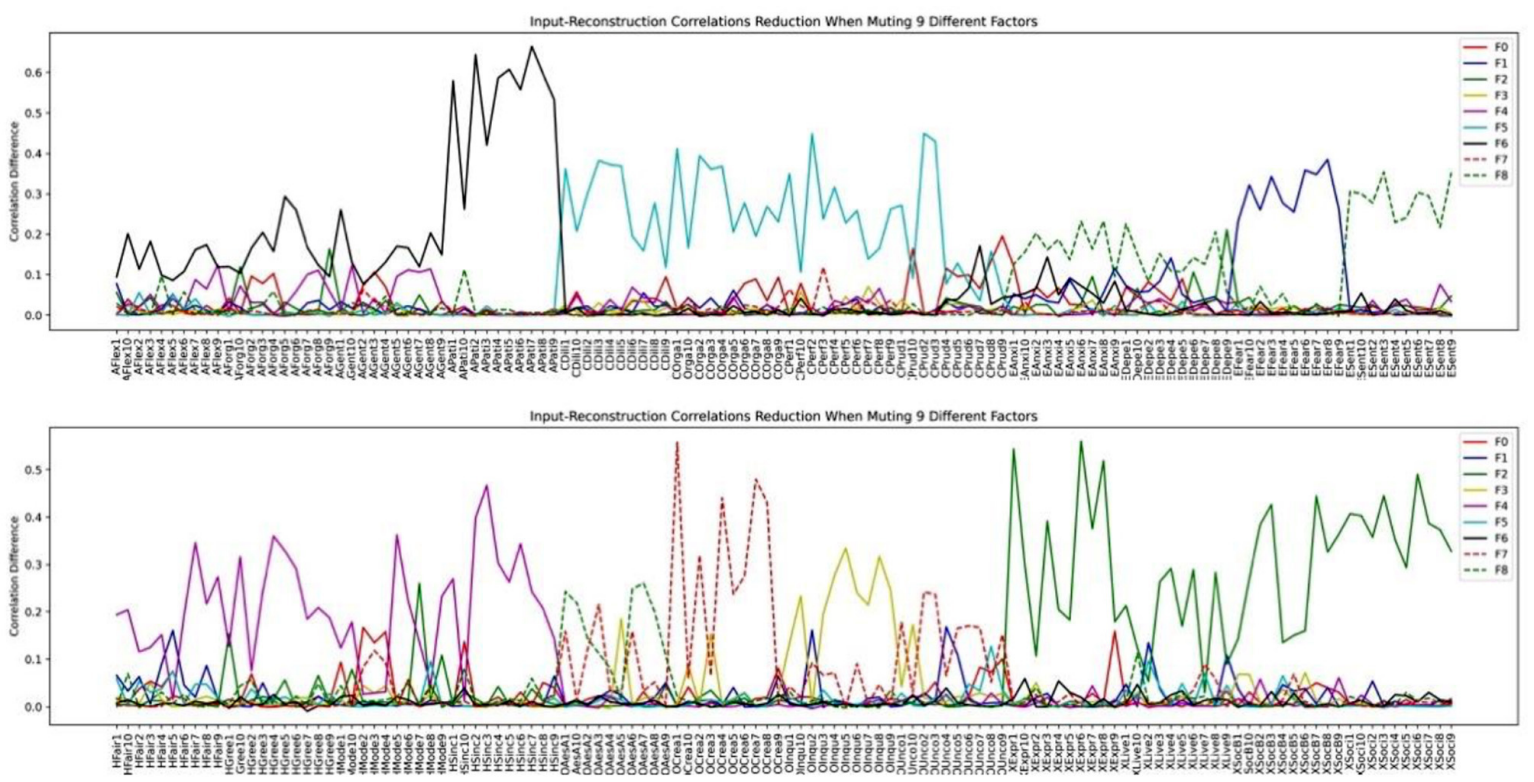
HEXACO factor-personality variable association when 9 latent factors are assumed in VAE.

**Figure 15:**
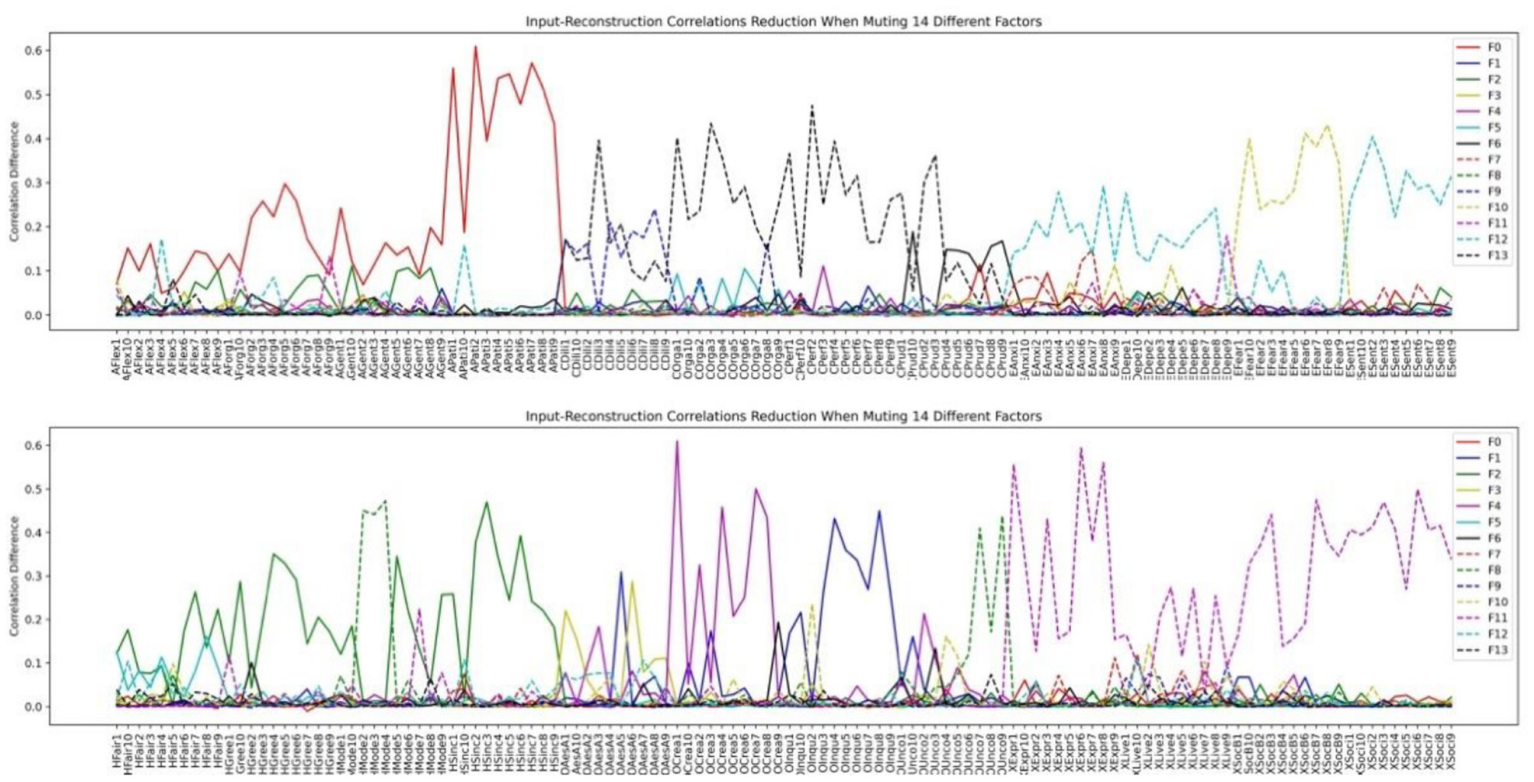
HEXACO factor-personality variable association when 14 latent factors are assumed in VAE.

We then inspected the factor structure through the input-reconstruction correlation reduction plots when we set the number of bottleneck layer nodes to 9 and 14, respectively in Figures 14, 15.

Comparing Figures 14, 15, we can see that the factor structures are mostly the same. The top panel consists of 4 factors: Agreeableness, Conscientiousness, Thrill-seeking (the Fearfulness facet of Emotionality), Emotionality (All facets of Emotionality except the Fearfulness facet). The bottom panel consists of 4 major factors: Machiavellianism (All Humility-Honesty facets except two Modesty variables), Creativity (The Creativity facet of Openness to Experience), Inquisitiveness (The Inquisitiveness facet of Openness to Experience), and Extraversion. Humility (Two Modesty facet variables in Humility-Honesty plus several Unconventionality facet variables in Openness to Experience) appeared as a stable factor in both our VAE stability analysis and the 14-factor analysis (dashed green line in Figure 15).

Unlike the LFA analysis which keeps on fractionating as we increase the number of latent factors, VAE does not change the factor structure significantly and increasing the number of factors beyond the nine prominent factors added noise-like factors, such as F3, F5, F6, F7, F8 in Figure 15 (We define a factor as noise like factors when the maximum reduction on correlations is less than 0.1 and there are fewer than 5 items under the factor. Future research shall be conducted in terms of how to best chose these thresholds.). We can see that inspecting the limit on the number significant factors is a viable method for determining the number of factors to be extracted. We term this as the Significant Factor Limit Search (SFLS) method.

We conclude that by using VAE, we can determine the factor structure directly when a large pool of personality variables exists. Facet level analysis is not necessary since no significant factors can be discovered after the stable factors. Although we still cannot guarantee that the stability analysis or this SFLS method reports the true number of factors, at least in this case, the two types of analysis reported similar number of factors in HEXACO and Big 5. Unlike in LFA, the two types of analysis may return radically different number of factors especially when the set of analyzed personality variables are complex.

The zero-order correlations between the 9 discovered factors are listed in Table 4. The most significant correlations are highlighted in bold.

**Table 4:** Correlations between rotated factors in the IPIP HEXACO VAE analysis.

	**(1)**	**(2)**	**(3)**	**(4)**	**(5)**	**(6)**	**(7)**	**(8)**
(1) Inquisitiveness	1							
(2) Agreeableness	0.03	1						
(3) Extraversion	0	0.05^*^	1					
(4) Conscientiousness	–0.14^**^	0.05^*^	0.06^**^	1				
(5) Humility	0.05^*^	−0.01	0.05^*^	–0.06^**^	1			
(6) Machiavellianism	0.02	–0.09^**^	−0.05^*^	0.02	0.04^*^	1		
(7) Thrill-seeking	–0.09^**^	0.04^*^	0.04^*^	0.02	−0.04^*^	0.07^**^	1	
(8) Emotionality	0	0.05^*^	0.05^*^	−0.03	0.04^*^	−0.04^*^	0.08^**^	1
(9) Creativity	–0.07^**^	−0.03	0.06^**^	0.1	−0.02	0.02	−0.04^*^	0.03

*N = 3,756 testing samples*;

* *P < 0.05; Bold values and*

** *are indicate that P-value < 0.001*.

### Cross-Regional Study

There is no significant deviation in mean, standard deviation. The mean and standard deviation statistics are included in the Supplementary Material. We further studied the zero-order correlations between the 9 factors in different regions. Again, little variation was detected. We listed the zero-order correlations in West Europe and Asia in Tables 5, 6 as examples. Please see the Supplementary Material for the rest of the regions.

**Table 5:** Correlations between factors trained on North American samples and tested on West Europe samples.

	**(1)**	**(2)**	**(3)**	**(4)**	**(5)**	**(6)**	**(7)**	**(8)**
(1) Machiavellianism	1							
(2) Emotionality	0.08^**^	1						
(3) Thrill-seeking	0.05^*^	0.01	1					
(4) Conscientiousness	0.2	0.14^**^	−0.05^*^	1				
(5) Inquisitiveness	−0.12^**^	−0.11^**^	−0.03	−0.08^**^	1			
(6) Creativity	0.1	0.01	0	−0.07^**^	−0.02	1		
(7) Extraversion	−0.07^**^	0.06^*^	−0.04	−0.04^*^	0.21^**^	−0.18^**^	1	
(8) Agreeableness	0.05	0.14^**^	0.03	0.18^**^	−0.06^*^	0.04^*^	−0.13^**^	1
(9) Humility	−0.08^**^	0.03	0.08^**^	−0.08^**^	−0.04^*^	0.11^**^	−0.04^*^	−0.08^**^

*N = 2,607*;

* *P < 0.05; Bold values and*

** *are indicate that P-value < 0.001*.

**Table 6:** Correlations between factors trained on North American samples and tested on Asian Samples.

	**(1)**	**(2)**	**(3)**	**(4)**	**(5)**	**(6)**	**(7)**	**(8)**
(1) Machiavellianism	1							
(2) Emotionality	0.15^**^	1						
(3) Thrill-seeking	−0.07^*^	0	1					
(4) Conscientiousness	0.13^**^	0.12^**^	−0.01	1				
(5) Inquisitiveness	−0.09^*^	−0.12^**^	−0.02	−0.05	1			
(6) Creativity	0.06	0.02	0.09^*^	−0.04	−0.11^*^	1		
(7) Extraversion	−0.17^**^	0.02	0.06	0	0.16^**^	−0.09^*^	1	
(8) Agreeableness	−0.11^*^	0.08^*^	0.06	0.21^**^	−0.02	0.03	−0.12^**^	1
(9) Humility	−0.12^**^	0.11^*^	0.11^*^	−0.04	−0.02	0.11^*^	0.01	−0.07^*^

*N = 850*;

* *P < 0.05; Bold values and*

** *are indicate that P-value < 0.001*.

Note that there are significantly larger correlations in the cross-regional study. This difference is caused by a smaller number of training samples, which affected the convergence of the VAE algorithm. The cross-region study was aimed at verifying the consistency of factor statistics across different regions, the results cannot be directly compared to the results obtained by using training samples from all regions.

## Discussions

In this paper, we first investigated how VAE can be applied in exploring the factor structure in personality variables and how it compares to LFA. Past research showed that the personality model discovered via LFA must organize the scales at the factor and the facet level. When the number of investigated personality variables is large, LFA returns unstable factor structures as the number of assumed latent factors grows. LAF must cut a smaller number of factors such that the factors are generalizable. Yet, it is well-known that factor level representation alone is not sufficient and facet level information cannot be ignored when personality models are used for predicting various behaviors. In contrast, VAE returns stable factor structures even if the number of assumed latent factors is greater than the number of stable factors. Consequently, VAE can be applied to explore factor structures in a one-shot process given a pool of personality variables. A follow-up analysis at the facet level is not necessary and consequently, a lot of *ad-hoc* decisions can be avoided in the process.

This difference is due to VAE's inherent ability in dealing with non-linear data models. The fact that LFA cannot exhaust all useful information at the factor level is an indication of the non-linearity in the underlying personality model.

Note that VAE and LFA returned similar factor structures in the Big5 dataset. This shows that the 50 variables in the IPIP Big 5 dataset are mostly linearly related to the latent factors. On the other hand, the factor structures returned by VAE and LFA are very different in the more complicated HEXACO dataset, which indicates that the 240 variables in HEXACO are less linearly organized by the latent factors.

We aim to show that VAE is a viable analytical tool for exploratory factor analysis in this paper. The datasets we analyzed were collected using the IPIP Big 5 and IPIP HEXACO questionnaires for the purpose of comparison with LFA. We fully understand that the resulting factor models may not be comprehensive. There could still exist factors that the questionnaires have not covered, and self-reported data will limit the usefulness of the calculated model. Now, we will be able to extend the application of VAE to include various types of data in the future for constructing useful personality models.

For proper personality model development, VAE can be applied in a two-stage process: In stage one, the aim is to discover all essential and stable factors and select personality variables to represent all the factors properly. Comparing the stable factors found in the IPIP Big5 and the IPIP HEXACO analyses, we can see that similar factors can be discovered and aligned together even if the measured personality variables are not entirely identical. This suggests that in the future, we may perform a meta-analysis of multiple datasets and then pool all the discovered factors and their associated personality variables together for final analysis. In stage two, enough sample data should be collected for the pooled personality variables to construct a comprehensive personality model. Furthermore, it is anticipated that observer report data can be easily incorporated in the VAE framework as we can simply extend the input vector to include observer reported variables.

### Limitations of VAE

The main limitation of effective VAE application is the number of samples available for analysis. For example, when we applied VAE on a reduced dataset with 2,099 samples, the reported mean (std) of the input-reconstruction correlations was 0.61 (0.08), which is significantly smaller than 0.65 (0.08) when all 18,779 samples are used in the HEXACO analysis. This indicates that VAE cannot converge to a lower cost function value without enough samples. VAE analysis requires a significantly larger number of samples than those required in LFA.

The required number of samples should also scale with the number of input variables. For example, in the IPIP HEXACO analysis, when we reduce the number of input variables to 79, the algorithm returns a much higher variable-wise mean correlation of 0.72 (0.08) than the case with 240 variables at 0.65 (0.08). However, if we reduce the number of input variables, some of the discovered factors will not be adequately represented by a group of personality variables just like the case of Big 5 dataset analysis. The pruning of the input items should be done carefully such that all factors are well represented.

We should balance the need to include more personality variables with the need to collect more samples, so that the VAE algorithm can converge properly. If the goal is to discover stable factors, then the average congruence score, the number of stable factors, and the reliability of the discovered factors can be used as guiding metrics in determining if enough samples have been included. One should evaluate if reducing the number of samples will affect the constructed model in all these statistics. If increasing the number of samples doesn't change the set of discovered stable factors, then the number of samples is enough.

If an accurate encoder is required, the guiding metric should be the mean and standard deviation of the input-reconstruction correlations. Enough samples should be included such that these measures can meet the requirement.

Another limitation of VAE is overfitting. To prevent this problem, besides a strict separation in the training testing dataset, we also monitor the loss function values calculated based on the training and the validation dataset. When the loss function value in the validation dataset does not decrease with the loss function in the training dataset, we stop the algorithm to prevent over-fitting.

While VAE-based factor analysis can be used to explore non-linear associations between latent factors and manifest personality variables, a common concern is the interpretability of the model. VAE does not have the equivalent measure of factor loading as in LFA. However, as we have demonstrated, the reduction of input-reconstruction correlations can be used as a substitute for examining factor-variable associations which returned similar results to that of factor loadings in LFA. Further research is needed to confirm its suitability as a measure for interpretating the discovered factors in VAE for practitioners to adopt it.

### Future Research

In VAE, since we do not have a reliability measure as in LFA, we cannot determine what is adequate for representing a factor. In LFA, the convention in the field has been that each factor is represented by 5–10 variables with high alpha reliability. However, it seems that these calculated measures are never enough and a link to the biological basis is required for the factors to be called “adequate” in the view of some researchers. Ultimately, we should test how well VAE constructed factor structures can be used for various behavior predictions in comparison to LFA.

Another area for extending the current research is to incorporate observer report data into personality model construction. The hurdle lies in the availability of large observer datasets since it is much more difficult and costly to collect observer ratings. Nevertheless, given the increasing availability of various data and the complicated data structure, VAE shall find broad applications in such areas.

## Data Availability Statement

The original contributions presented in the study are included in the article/Supplementary Material, further inquiries can be directed to the corresponding author/s. All code is made available on OSF (https://osf.io/6bf3w/).

## Ethics Statement

Ethical review and approval was not required for the study on human participants in accordance with the local legislation and institutional requirements. Written informed consent from the patients/participants was not required to participate in this study in accordance with the national legislation and the institutional requirements.

## Author Contributions

JZ came up with the idea of investigating personality model construction using deep learning methods, implemented the algorithm, and wrote the manuscript. YH identified variational autoencoder as the appropriate tool for the task and advised on the application of VAE to the problem. Both authors contributed to the article and approved the submitted version.

## Conflict of Interest

The authors declare that the research was conducted in the absence of any commercial or financial relationships that could be construed as a potential conflict of interest.

## Publisher's Note

All claims expressed in this article are solely those of the authors and do not necessarily represent those of their affiliated organizations, or those of the publisher, the editors and the reviewers. Any product that may be evaluated in this article, or claim that may be made by its manufacturer, is not guaranteed or endorsed by the publisher.
