# Supplementary material for: Exploring Factor Structures Using Variational Autoencoder in Personality Research
Source: Front Psychol. 2022 Aug 5;13:863926. doi: 10.3389/fpsyg.2022.863926 (PMC9388855; doi:10.3389/fpsyg.2022.863926)
Supplement: Supplementary file 1 [file Table_1.DOCX]

# **Supplementary File- Variational Autoencoder Constructs Better Personality Models Than Linear Factor Analysis**

In Supplementary Figure 1, we show the stable factor analysis result from the VAE analysis of the IPIP Big5 dataset when 9 bottleneck layer nodes are used:


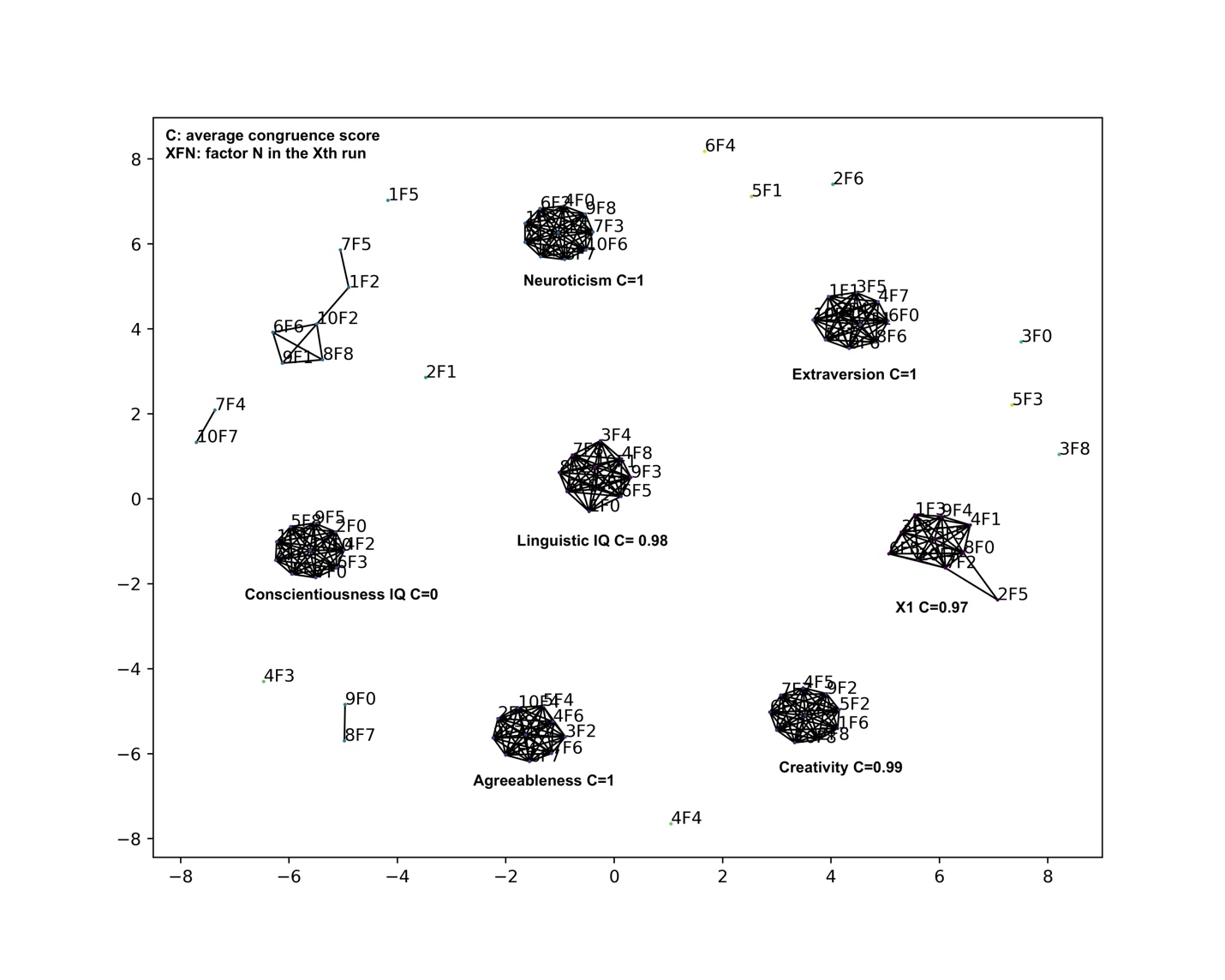


*Supplementary Figure 1: Clustering of factors from 10 VAE runs with 9 bottleneck layer nodes and 100 middle layer nodes in the Big 5 analysis*

We can see that compared to Figure 4 in the main paper, the seven identified stable factors are identical.

**Cross regional study based on the IPIP HEXACO dataset**

In Supplementary Table 1 and 2, we list the mean and the standard deviation of the derived factors from the VAE HEAXCO analysis across different regions.

| Supplementary Table 1  Mean of the derived factors across different regions | | | | | | | | | | | | |  |
| --- | --- | --- | --- | --- | --- | --- | --- | --- | --- | --- | --- | --- | --- |
|  | North America | | South America | | West Europe | | Asia | | Oceania | | East Europe | |  |
| (1) Machiavellianism | | 3.66 | | 3.8 | | 3.7 | | 4.02 | | 3.77 | | 3.88 | |
| (2) Emotionality | | 4.4 | | 4.4 | | 4.25 | | 4.45 | | 4.26 | | 4.16 | |
| (3) Thrill-seeking | | 4.08 | | 4.15 | | 4.03 | | 4.23 | | 4.05 | | 4.07 | |
| (4) Conscientiousness | | 4.31 | | 4.45 | | 4.23 | | 4.38 | | 4.24 | | 4.15 | |
| (5) Linguistic IQ | | 4.14 | | 4.28 | | 4.16 | | 4.26 | | 4.17 | | 4.25 | |
| (6) Creativity | | 4.79 | | 4.82 | | 4.72 | | 4.76 | | 4.71 | | 4.74 | |
| (7) Extraversion | | 3.98 | | 4.04 | | 3.98 | | 4.06 | | 3.99 | | 3.99 | |
| (8) Agreeableness | | 4.2 | | 4.23 | | 4.16 | | 4.25 | | 4.17 | | 4.08 | |
| (9) Humility | | 3.77 | | 3.79 | | 3.67 | | 4.05 | | 3.75 | | 3.63 | |
| Mean of mean | | 4.15 | | 4.22 | | 4.1 | | 4.27 | | 4.12 | | 4.11 | |
| Number of samples | | 3522 | | 692 | | 2670 | | 850 | | 1005 | | 400 | |

| Supplementary Table 2  Standard deviation of the derived factors across different regions | | | | | | | | | | | | |  |
| --- | --- | --- | --- | --- | --- | --- | --- | --- | --- | --- | --- | --- | --- |
|  | North America | | South America | | West Europe | | Asia | | Oceania | | East Europe | |  |
| (1) Machiavellianism | | 1.73 | | 1.8 | | 1.76 | | 1.74 | | 1.74 | | 1.76 | |
| (2) Emotionality | | 1.65 | | 1.66 | | 1.7 | | 1.67 | | 1.68 | | 1.77 | |
| (3) Thrill-seeking | | 1.72 | | 1.72 | | 1.72 | | 1.71 | | 1.72 | | 1.76 | |
| (4) Conscientiousness | | 1.59 | | 1.59 | | 1.64 | | 1.62 | | 1.59 | | 1.67 | |
| (5) Linguistic IQ | | 1.61 | | 1.68 | | 1.61 | | 1.65 | | 1.62 | | 1.63 | |
| (6) Creativity | | 1.27 | | 1.27 | | 1.34 | | 1.33 | | 1.32 | | 1.36 | |
| (7) Extraversion | | 1.82 | | 1.81 | | 1.81 | | 1.78 | | 1.78 | | 1.75 | |
| (8) Agreeableness | | 1.8 | | 1.82 | | 1.79 | | 1.78 | | 1.75 | | 1.82 | |
| (9) Humility | | 1.7 | | 1.73 | | 1.68 | | 1.77 | | 1.69 | | 1.71 | |
| Mean of std | | 1.65 | | 1.68 | | 1.67 | | 1.67 | | 1.65 | | 1.69 | |
| Number of samples | | 3522 | | 692 | | 2670 | | 850 | | 1005 | | 400 | |

We can see that surprisingly, the Creativity factor has the smallest standard deviation across the regions. Also, the mean of standard deviation decreases as the number of samples increases. Overall, the statistics are highly consistent across different regions.

In supplementary table 3, we list the zero order correlations between all factors across different regions

| Supplementary Table 3: Zero order correlations between factors in different regions | | | | | | | | | | | | | | | | |  |  |
| --- | --- | --- | --- | --- | --- | --- | --- | --- | --- | --- | --- | --- | --- | --- | --- | --- | --- | --- |
| **North America** |  | |  | |  | |  | |  | |  | |  | |  | | |  |
|  | | (1) | | (2) | | (3) | | (4) | | (5) | | (6) | | (7) | | (8) | | |
| (1) Machiavellianism | | 1 | |  | |  | |  | |  | |  | |  | |  | | |
| (2) Emotionality | | 0.12** | | 1 | |  | |  | |  | |  | |  | |  | | |
| (3) Thrill-seeking | | 0.04* | | 0.06** | | 1 | |  | |  | |  | |  | |  | | |
| (4) Conscientiousness | | 0.12** | | 0.04* | | -0.02 | | 1 | |  | |  | |  | |  | | |
| (5) Linguistic IQ | | -0.08** | | -0.08* | | -0.03* | | -0.04* | | 1 | |  | |  | |  | | |
| (6) Creativity | | 0.08** | | -0.03* | | 0 | | -0.03* | | 0.03* | | 1 | |  | |  | | |
| (7) Extraversion | | -0.03* | | 0.1** | | 0.01 | | -0.05* | | 0.2** | | -0.15** | | 1 | |  | | |
| (8) Agreeableness | | 0 | | 0.08** | | -0.03* | | 0.18** | | -0.03* | | 0.1** | | -0.09** | | 1 | | |
| (9) Humility | | -0.07** | | 0 | | 0.07** | | -0.02 | | -0.08** | | 0.06* | | -0.03* | | -0.06* | | |
| N=3522. * r>0.028 P-value<0.05; ** r>0.06, P-value<0.001 | | | | | | | | | | | | | | | | | | |
| **South America** |  | |  | |  | |  | |  | |  | |  | |  | | |  |
|  | (1) | | (2) | | (3) | | (4) | | (5) | | (6) | | (7) | | (8) | | |  |
| (1) Machiavellianism | 1 | |  | |  | |  | |  | |  | |  | |  | | |  |
| (2) Emotionality | 0.14** | | 1 | |  | |  | |  | |  | |  | |  | | |  |
| (3) Thrill-seeking | -0.09* | | -0.04 | | 1 | |  | |  | |  | |  | |  | | |  |
| (4) Conscientiousness | 0.1* | | 0.1* | | 0.09* | | 1 | |  | |  | |  | |  | | |  |
| (5) Linguistic IQ | -0.15** | | -0.18** | | 0.05 | | -0.02 | | 1 | |  | |  | |  | | |  |
| (6) Creativity | 0.09* | | 0.03 | | 0.01 | | 0.09* | | -0.06 | | 1 | |  | |  | | |  |
| (7) Extraversion | -0.01 | | 0.04 | | -0.02 | | 0 | | 0.1* | | -0.16** | | 1 | |  | | |  |
| (8) Agreeableness | 0.04 | | 0.17** | | 0.02 | | 0.17** | | -0.12** | | 0.09* | | -0.05 | | 1 | | |  |
| (9) Humility | -0.12** | | 0.07* | | 0.09* | | -0.09* | | -0.04 | | 0.01 | | -0.01 | | -0.08* | | |  |
| \| N=692. * r>0.06 P-value<0.05; ** r>0.11, P-value<0.001 \| \| --- \| | | | | | | | | | | | | | | | | |  |  |
| **West Europe** |  | |  | |  | |  | |  | |  | |  | |  | | |  |
|  | (1) | | (2) | | (3) | | (4) | | (5) | | (6) | | (7) | | (8) | | |  |
| (1) Machiavellianism | 1 | |  | |  | |  | |  | |  | |  | |  | | |  |
| (2) Emotionality | 0.08** | | 1 | |  | |  | |  | |  | |  | |  | | |  |
| (3) Thrill-seeking | 0.05* | | 0.01 | | 1 | |  | |  | |  | |  | |  | | |  |
| (4) Conscientiousness | 0.2** | | 0.14** | | -0.05* | | 1 | |  | |  | |  | |  | | |  |
| (5) Linguistic IQ | -0.12** | | -0.11** | | -0.03 | | -0.08** | | 1 | |  | |  | |  | | |  |
| (6) Creativity | 0.1** | | 0.01 | | 0 | | -0.07** | | -0.02 | | 1 | |  | |  | | |  |
| (7) Extraversion | -0.07** | | 0.06* | | -0.04* | | -0.04* | | 0.21** | | -0.18** | | 1 | |  | | |  |
| (8) Agreeableness | 0.05* | | 0.14** | | 0.03 | | 0.18** | | -0.06* | | 0.04* | | -0.13** | | 1 | | |  |
| (9) Humility | -0.08** | | 0.03 | | 0.08** | | -0.08** | | -0.04* | | 0.11** | | -0.04* | | -0.08** | | |  |
| N=2670. * r>0.032 P-value<0.05; ** r>0.07, P-value<0.001 | | | | | | | | | | | | | | | | |  |  |
| **Asia** |  | |  | |  | |  | |  | |  | |  | |  | | |  |
|  | (1) | | (2) | | (3) | | (4) | | (5) | | (6) | | (7) | | (8) | | |  |
| (1) Machiavellianism | 1 | |  | |  | |  | |  | |  | |  | |  | | |  |
| (2) Emotionality | 0.15** | | 1 | |  | |  | |  | |  | |  | |  | | |  |
| (3) Thrill-seeking | -0.07* | | 0 | | 1 | |  | |  | |  | |  | |  | | |  |
| (4) Conscientiousness | 0.13** | | 0.12* | | -0.01 | | 1 | |  | |  | |  | |  | | |  |
| (5) Linguistic IQ | -0.09* | | -0.12* | | -0.02 | | -0.05 | | 1 | |  | |  | |  | | |  |
| (6) Creativity | 0.06* | | 0.02 | | 0.09* | | -0.04 | | -0.11* | | 1 | |  | |  | | |  |
| (7) Extraversion | -0.17** | | 0.02 | | 0.06* | | 0 | | 0.16** | | -0.09* | | 1 | |  | | |  |
| (8) Agreeableness | -0.11* | | 0.08* | | 0.06* | | 0.21** | | -0.02 | | 0.03 | | -0.12** | | 1 | | |  |
| (9) Humility | -0.12* | | 0.11* | | 0.11** | | -0.04 | | -0.02 | | 0.11* | | 0.01 | | -0.07* | | |  |
| N=850. * r>0.055 P-value<0.05; ** r>0.13, P-value<0.001 | | | | | | | | | | | | | | | | |  |  |
| **Oceania** |  | |  | |  | |  | |  | |  | |  | |  | | |  |
|  | (1) | | (2) | | (3) | | (4) | | (5) | | (6) | | (7) | | (8) | | |  |
| (1) Machiavellianism | 1 | |  | |  | |  | |  | |  | |  | |  | | |  |
| (2) Emotionality | 0.06* | | 1 | |  | |  | |  | |  | |  | |  | | |  |
| (3) Thrill-seeking | 0.03 | | 0.03 | | 1 | |  | |  | |  | |  | |  | | |  |
| (4) Conscientiousness | 0.14** | | 0.06* | | -0.09* | | 1 | |  | |  | |  | |  | | |  |
| (5) Linguistic IQ | -0.15** | | -0.12** | | -0.07* | | -0.07* | | 1 | |  | |  | |  | | |  |
| (6) Creativity | 0.09 | | 0 | | -0.01 | | -0.06* | | -0.09* | | 1 | |  | |  | | |  |
| (7) Extraversion | -0.01 | | 0.04 | | 0.02 | | -0.02 | | 0.21** | | -0.15** | | 1 | |  | | |  |
| (8) Agreeableness | 0.01 | | 0.07* | | -0.03 | | 0.14** | | -0.07* | | 0.14** | | -0.15** | | 1 | | |  |
| (9) Humility | -0.1** | | 0 | | 0.06* | | -0.06* | | -0.08* | | 0.03 | | -0.05 | | -0.06* | | |  |
| N=1005. * r>0.051 P-value<0.05; ** r>0.097, P-value<0.001 | | | | | | | | | | | | | | | | |  |  |
| **East Europe** |  | |  | |  | |  | |  | |  | |  | |  | | |  |
|  | (1) | | (2) | | (3) | | (4) | | (5) | | (6) | | (7) | | (8) | | |  |
| (1) Machiavellianism | 1 | |  | |  | |  | |  | |  | |  | |  | | |  |
| (2) Emotionality | 0.28** | | 1 | |  | |  | |  | |  | |  | |  | | |  |
| (3) Thrill-seeking | -0.06 | | 0.06 | | 1 | |  | |  | |  | |  | |  | | |  |
| (4) Conscientiousness | 0.28** | | 0.11* | | 0.03 | | 1 | |  | |  | |  | |  | | |  |
| (5) Linguistic IQ | -0.19** | | -0.23** | | -0.05 | | -0.16** | | 1 | |  | |  | |  | | |  |
| (6) Creativity | 0.15* | | 0.05 | | 0.07 | | 0.04 | | -0.19** | | 1 | |  | |  | | |  |
| (7) Extraversion | -0.15* | | 0.1 | | -0.08 | | -0.1* | | 0.15* | | -0.18** | | 1 | |  | | |  |
| (8) Agreeableness | 0.09* | | 0.09* | | 0.07 | | 0.33** | | -0.17** | | 0.17** | | -0.28** | | 1 | | |  |
| (9) Humility | -0.07 | | 0.09* | | 0.12* | | -0.02 | | -0.18** | | 0.1 | | 0.03 | | -0.08 | | |  |
| N=400. * r>0.082 P-value<0.05; ** r>0.154, P-value<0.001 | | | | | | | | | | | | | | | | |  |  |

We can see that there are differences in the correlations between some factors across different regions but most of the significant correlations are reproduced across different regions.
